# Exceptional Longevity Modifying Allele *APOE2* Promotes DNA Signaling Pathways Resisting Cellular Senescence in Human Neurons

**DOI:** 10.1111/acel.70494

**Published:** 2026-05-08

**Authors:** Cristian Gerónimo‐Olvera, Stephen M. Scheeler, Carlos Galicia Aguirre, Genesis Vega‐Hormazabal, Daniela Garcia, Long Wu, Natalia Murad, Kevin Schneider, Kenneth A. Wilson, Nikola T. Markov, Sicheng Song, Jesse Simons, Akos A. Gerencser, Emily Parlan, Sean D. Mooney, Eric Verdin, Judith Campisi, Tara E. Tracy, David Furman, Simon Melov, Lisa M. Ellerby

**Affiliations:** ^1^ Buck Institute for Research on Aging Novato California USA; ^2^ University of Southern California, Leonard Davis School of Gerontology Los Angeles California USA; ^3^ Department of Biomedical Informatics and Medical Education University of Washington Seattle Washington USA

**Keywords:** aging, *APOE2*, DNA damage, exceptional longevity, neurons, senescence

## Abstract

Genome‐wide association studies (GWAS) have identified *APOE2* allele as linked to exceptional longevity, with carriers exhibiting a reduced risk of Alzheimer's disease (AD). Apolipoprotein E (APOE), a glycoprotein involved in lipid transport, has three major alleles. However, alterations in lipid metabolism alone do not fully explain APOE2's protective effects. In contrast, *APOE4* is the strongest genetic risk factor for AD. To investigate how APOE2 promotes neuronal longevity and confers neuroprotection, we generated human isogenic *APOE* iPSC‐derived models of both inhibitory GABAergic and excitatory neurons. In GABAergic neurons, *APOE* alleles differentially influenced endogenous DNA damage, DNA repair, and neuronal motility. Single‐cell RNA sequencing revealed *APOE4*‐specific gene expression signatures associated with AD, whereas *APOE2* GABAergic neurons were enriched for DNA repair and signaling pathways. Consistent with this, *APOE2* neurons exhibited significantly lower levels of DNA damage. *APOE4* GABAergic neurons exhibit increased expression of repetitive ribosomal RNA, which is associated with DNA damage and cellular senescence. To determine whether the effects extended to excitatory neurons, we used a separate human model of Ngn2‐induced glutamatergic neurons, and found that *APOE2* excitatory neurons were more resistant to cellular senescence and DNA damage than isogenic *APOE3* and *APOE4* neurons. Similarly, we found human APOE2‐targeted replacement mice exhibited less nucleolar enlargement and increased nuclear Lamin A/C, Hmgb1, and H3K9me3 compared to *APOE4* counterparts. Together, our findings identify DNA repair and suppression of senescence‐associated processes as key mechanisms by which APOE2 is associated with neuronal resilience, providing mechanistic insight into its association with exceptional longevity and protection against AD.

## Graphical abstract

Neurons expressing APOE2 were more resistant to endogenous DNA damage, activated transcriptional signaling pathways associated with DNA repair, and were resilient to stress‐induced DNA damage and cellular senescence. In contrast, *APOE4* neurons exhibited elevated expression of rRNA repetitive elements and were prone to becoming senescent.

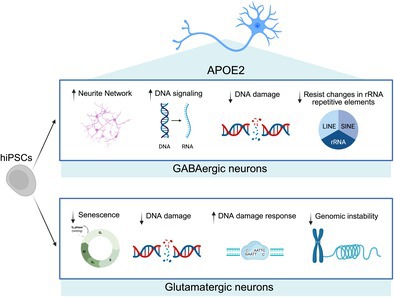

**Abbreviations**
AD: Alzheimer's disease
APOE: apolipoprotein E
Doxo: doxorubicin
GO: Gene Ontology
GWAS: Genome‐wide association studies
iPSC: induced pluripotent stem cell
IR: irradiation
RNAseq: RNA sequencing
rRNA: ribosomal RNA
scRNAseq: single‐cell RNA sequencing

## 1 Introduction

Major challenges remain in understanding the biological basis of human longevity and identifying strategies to improve healthspan. Although environmental factors influence aging, healthy aging has a significant heritable component. Among the best‐validated longevity‐associated genes is apolipoprotein E2 (*APOE2*). A genome‐wide association study (GWAS) involving 2118 nonagenarian siblings across 11 European countries identified four chromosomal regions associated with familial longevity, including the *APOE* gene locus (Beekman et al. 2013). Subsequent studies in centenarians and semi‐centenarians from Spain, Italy, and Japan revealed a consistent positive association between the *APOE* ε2 allele and exceptional longevity, particularly in the Italian and Japanese cohorts. In the Italian study, the APOE *ε2* allele was also associated with healthier aging (Garatachea et al. 2014). A larger cohort study confirmed that carrying a single copy of APOE *ε2* significantly increased the likelihood of achieving advanced age (Sebastiani et al. 2019). In contrast, all studies agreed that the *APOE ε4* allele substantially reduces the chance of achieving exceptional longevity across various ethnic and geographic groups while also increasing mortality risk (Beekman et al. 2013; Sebastiani et al. 2019; Shinohara et al. 2020). Despite strong epidemiological evidence linking APOE2 to both longevity and reduced AD risk, the cellular mechanisms by which APOE2 promotes neuronal resilience remain poorly defined.

Aging is the main risk factor for neurodegenerative diseases, including Alzheimer's disease (AD)—the leading cause of dementia and a major contributor to mortality in the elderly. Although several genetic risk factors for late‐onset AD have been identified, *APOE ε4* remains the strongest, increasing AD risk by ~fourfold with one allele and ~14‐fold with two alleles relative to *APOE ε3* homozygosity (Bertram and Tanzi 2009; Kamboh et al. 2012). In addition, *APOE4* is associated with earlier onset, elevated amyloid plaque burden, and enhanced tau phosphorylation in both human and animal studies (Arnaud et al. 2022; Blanchard et al. 2020; Seo et al. 2023; Shi et al. 2017). Conversely, homozygous *APOE2* carriers exhibit markedly reduced AD incidence, supporting a dual role for *APOE2* in promoting longevity and offering neuroprotection.

APOE is a plasma glycoprotein critical for lipid transport and metabolism (Mahley and Rall Jr. 2000). It facilitates the binding and cellular internalization of lipoprotein complexes. In the CNS, it serves as the principal cholesterol carrier between astrocytes and neurons, supporting energy supply, synaptic remodeling, and neuronal repair (Petegnief et al. 2001). APOE exists in three isoforms—APOE2 (Cys112, Cys158), APOE3 (Cys112, Arg158), and APOE4 (Arg112, Arg158)—each differing by two amino acids. These small differences result in distinct receptor binding profiles and lipid processing, contributing to isoform‐specific roles in aging and disease (Ang et al. 2008). While astrocytes are the main producers of APOE in the CNS, neurons also express APOE under conditions of stress and aging (Blumenfeld et al. 2024; Koutsodendris et al. 2023). Furthermore, neuronal and astrocytic expression of APOE4 may independently drive AD pathogenesis, possibly through mechanisms such as increased neuronal hyperactivity, loss of inhibitory tone, and inflammation (Nuriel et al. 2017; Zalocusky et al. 2021).

Aging leads to the gradual accumulation of senescent cells that disrupt tissue homeostasis and drive age‐related disorders. Although neurons are post‐mitotic, they are susceptible to stressors that trigger a neuronal senescence‐like phenotype (e.g., DNA damage, mitochondrial dysfunction, and proteostasis failure). Despite the central role of senescence in aging and AD, direct links between APOE isoforms and neuronal senescence have not been established. A recent study using human induced pluripotent (iPSC)‐derived neurons found that APOE4 promotes synaptic gene dysregulation, increasing synapse number and Aβ42 secretion compared to isogenic *APOE3* neurons (Lin et al. 2018).

Given this background, we sought to evaluate the contribution of the *APOE* genotype to neuronal aging and vulnerability. Using bulk and single‐cell RNA‐sequencing (RNA‐seq) of iPSC‐derived GABAergic neurons, we identified genotype‐dependent differences in gene expression and DNA damage pathways. Notably, *APOE2* GABAergic neurons showed upregulated DNA repair signaling, and *APOE4* neurons exhibited increased synaptic gene expression, DNA damage, and altered cell motility. In a separate model of Ngn2‐induced glutamatergic neurons, *APOE2* neurons resisted genotoxic stress and were less prone to acquiring a senescent‐like phenotype than isogenic *APOE3* and *APOE4* neurons. Notably, recombinant APOE2 conferred *APOE4* glutamatergic neurons protection against irradiation‐induced DNA damage. Similarly, analysis of hippocampal tissue of human *APOE knock‐in* mice demonstrated that *APOE2* mice displayed features of healthier brain aging relative to *APOE3* and *APOE4* mice. These findings suggest a protective role of APOE2 in maintaining neuronal integrity through enhanced DNA repair and senescence resistance. These key mechanisms may underlie APOE2's association with longevity and reduced AD risk.

## 2 Results

### 2.1 Neurite Network and Movement Are Greater in *APOE2* Than *APOE4* GABAergic Neurons

**FIGURE 1:**
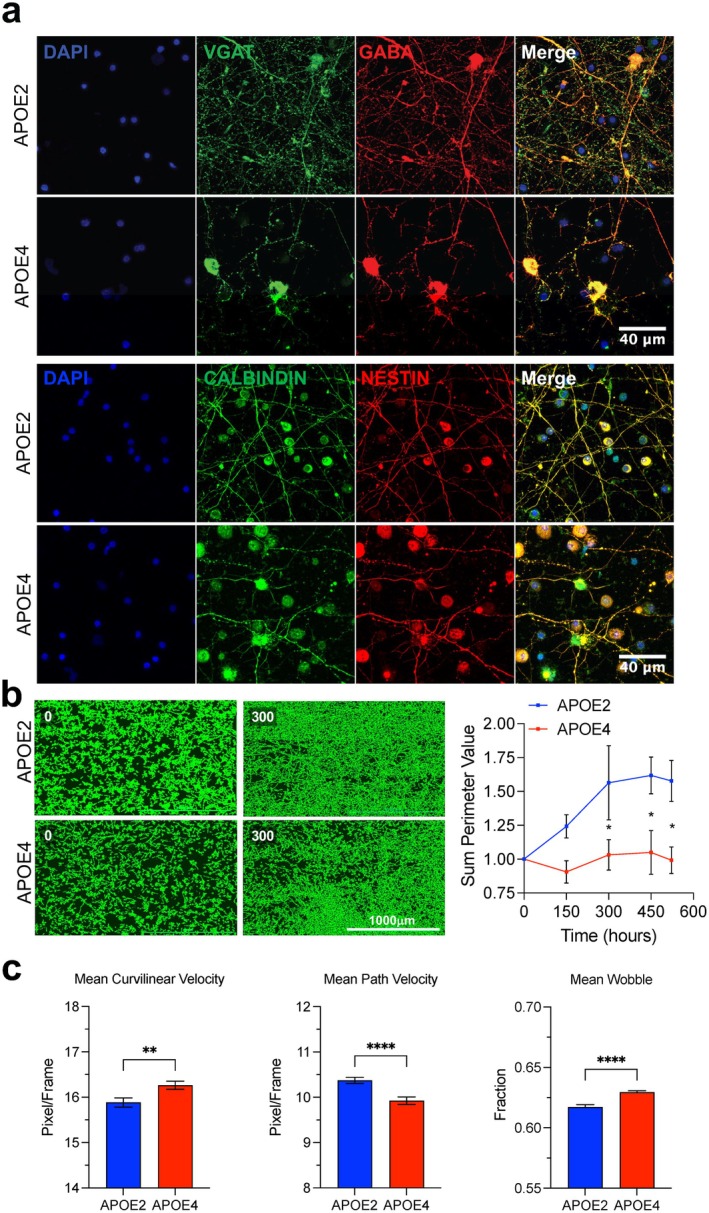
*APOE2* neurons exhibit increased neurite network complexity and improved motility compared to *APOE4* neurons. (a) Representative immunocytochemistry images of *APOE2* and *APOE4* GABAergic neurons stained with antibodies against VGAT (green), GABA (red), Calbindin (green), and Nestin (red). Nuclei were counterstained with DAPI (blue). Scale bar, 40 μm. (b) Representative neurite perimeter networks of *APOE* GABAergic neurons. Graph represents mean ± SEM, *n* = 3. Two‐way repeated measures ANOVA followed by Tukey's post hoc correction. *p < 0.05. Scale bar, 1000 μm. (c) Quantification of cell body motility parameters, including curvilinear velocity, average path velocity, and wobble. Bars represent mean ± SEM; *n* = 60, *t*‐test, **p < 0.01, ****p < 0.0001.

To determine how APOE2 contributes to neuronal resilience in inhibitory neurons, we differentiated human female isogenic iPSCs carrying homozygous *APOE2* or *APOE4* alleles into GABAergic neurons. Because interneuron dysfunction is implicated early in AD and APOE4 expression in interneurons suggests vulnerability to synaptic deficits and connectivity, we focused on this neuronal subtype vulnerability in neuronal connectivity (Knoferle et al. 2014; Najm et al. 2019). Immunocytochemistry confirmed robust expression of VGAT (vesicular inhibitory amino acid transporter) and GABA (γ‐aminobutyric acid)—markers of neurotransmitter synthesis and vesicular transport (Chaudhry et al. 1998)—as well as the neuronal markers CALBINDIN and NESTIN (Kojetin et al. 2006; Steinert et al. 1999) (Figure 1a). In agreement with previous reports (Brecht et al. 2004; Knoferle et al. 2014; Najm et al. 2019), *APOE4* neurons exhibited a pronounced loss of GABAergic neurite network density, relative to *APOE2* neurons, over a 21‐day culture period (Figure 1b). Quantitative live‐cell imaging revealed a significantly decreased neurite complexity in *APOE4* neurons at 300, 450, and 600 h of differentiation. Beyond neurite architecture, genotype‐specific alterations in neuronal motility were observed. Across 21 days of live imaging, *APOE4* neurons displayed increased mean curvilinear velocity but reduced path velocity, along with greater wobble amplitude compared to *APOE2* neurons (Figure 1c, Figure S1a). These findings reveal that *APOE2* and *APOE4* GABAergic neurons exhibit distinct cellular phenotypes, highlighting divergent effects of *APOE* genotype on inhibitory neuron structure and dynamics.

### 2.2 Transcriptomic Analysis of GABAergic Neurons Has Increased DNA Damage Response in *APOE2* Neurons

**FIGURE 2:**
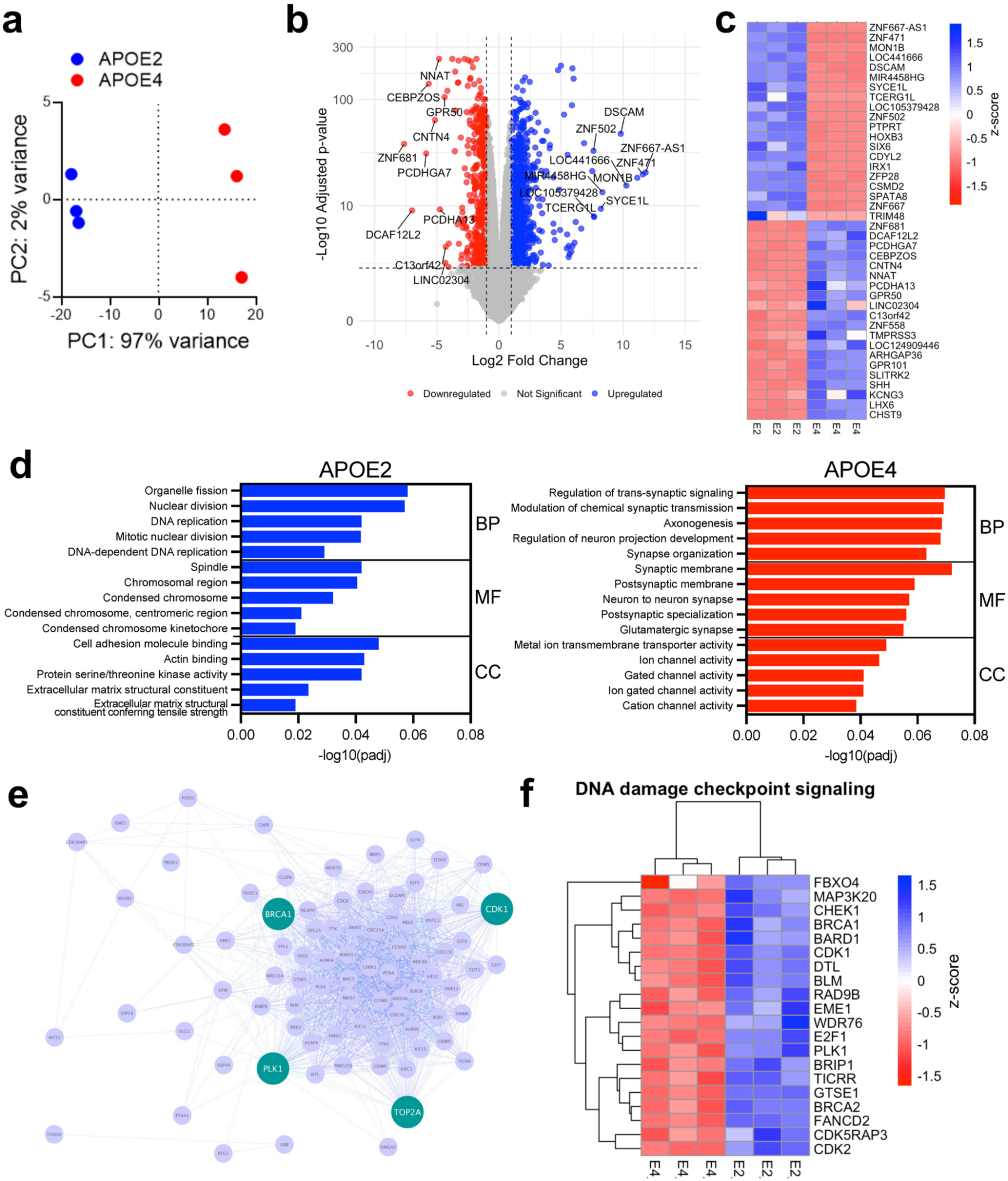
Transcriptomic analysis of isogenic *APOE2* and *APOE*4 GABAergic neurons reveals enrichment of DNA damage signaling pathways in *APOE2* neurons. (a) Bulk RNA‐seq analysis of *APOE2* and *APOE4* GABAergic neurons. The principal component analysis (PCA) plot shows distinct clustering by genotype (*n* = 3). (b) Volcano plot showing DEGs between *APOE2* and *APOE4*, highlighting the top 10 most upregulated and downregulated genes. (c) Heatmap of the top 20 upregulated and downregulated genes ranked by Log_2_ fold‐change, *p*adj < 0.05. (d) Gene Ontology (GO) enrichment analysis of RNA‐seq data showing significantly enriched biological process (BP), molecular function (MF), and cellular component (CC) terms. (e) Key hub analysis showing genes associated with DNA damage checkpoint signaling, including *BRCA1*, *CDK1*, *PLK1*, and *TOP2A*. *BRCA1* is involved in the repair of double‐strand breaks, *CDK1* is an effector kinase that responds to DNA damage, *PLK1* responds to DNA damage to maintain chromosome structure, and *TOP2A* helps regulate gene expression in relation to DNA damage and double‐stranded breaks. (f) Heatmap of 20 genes involved in DNA damage checkpoint signaling, comparing expression between *APOE2* and *APOE4* neurons.

To understand the distinct cellular phenotypes and underlying molecular mechanisms of the APOE isoforms, we performed an unbiased transcriptomic analysis of *APOE2* and *APOE4* GABAergic neurons. Principal component analysis (PCA) showed that the *APOE2* and *APOE4* GABAergic neurons segregated into distinct clusters (Figure 2a). We identified 1403 differentially expressed genes (DEGs) between *APOE2* and *APOE4* neurons (Figure 2b,c, Table S1). *APOE* mRNA was expressed in both genotypes (Figure S1b).

Among the most upregulated genes (*APOE2* vs. *APOE4*) were *DSCAM* (Down syndrome cell adhesion molecule), which regulates neuronal delamination (Arimura et al. 2020) and locomotion (Lemieux et al. 2021), and *CSMD2* (CUB and Sushi multidomains 2), which is involved in the development and maintenance of dendrites, synapses, and brain structure (Gutierrez et al. 2019). Neuronatin (*NNAT*), a gene upregulated in AD and associated with spine loss and endoplasmic reticulum calcium overload (Zou et al. 2022), was among the most downregulated genes in *APOE2* neurons. Contactin‐4 (*CNTN4*), involved in cell adhesion, APP function, and AD (Bamford et al. 2024), was also reduced in *APOE2* neurons (Figure 2b,c).

Gene Ontology (GO) analysis revealed that genes upregulated in *APOE2* neurons were significantly enriched for pathways related to DNA damage response and repair, whereas genes upregulated in the *APOE4* genotype were enriched for pathways involving synaptic activity, axonogenesis, and ion channel function (Figure 2d; Table S1). Network analysis of the DNA repair signaling pathway identified *BRCA1* (breast cancer 1), *CDK1* (cyclin‐dependent kinase 1), *PLK1* (polo‐like kinase 1), and *TOP2A* (DNA topoisomerase II alpha) as central hub genes in the *APOE2* GABAergic neurons (Figure 2e). A heatmap of the DNA damage checkpoint genes further confirmed upregulation of factors involved in DNA repair, damage resistance, and genomic stability, including *BLM* (Bloom syndrome recQ‐like helicase), *RAD9B* (checkpoint clamp component B), *BRCA1*, and *PLK1* (Figure 2f) in *APOE2* GABAergic neurons. Together, these results indicate allele‐specific transcriptional programs, with *APOE2* associated with enhanced DNA damage response mechanisms, and *APOE4* neurons biased toward synaptic signaling programs.

### 2.3 Single‐Cell RNA‐Seq Shows Unique Populations of GABAergic Neurons Relative to Genotype

**FIGURE 3:**
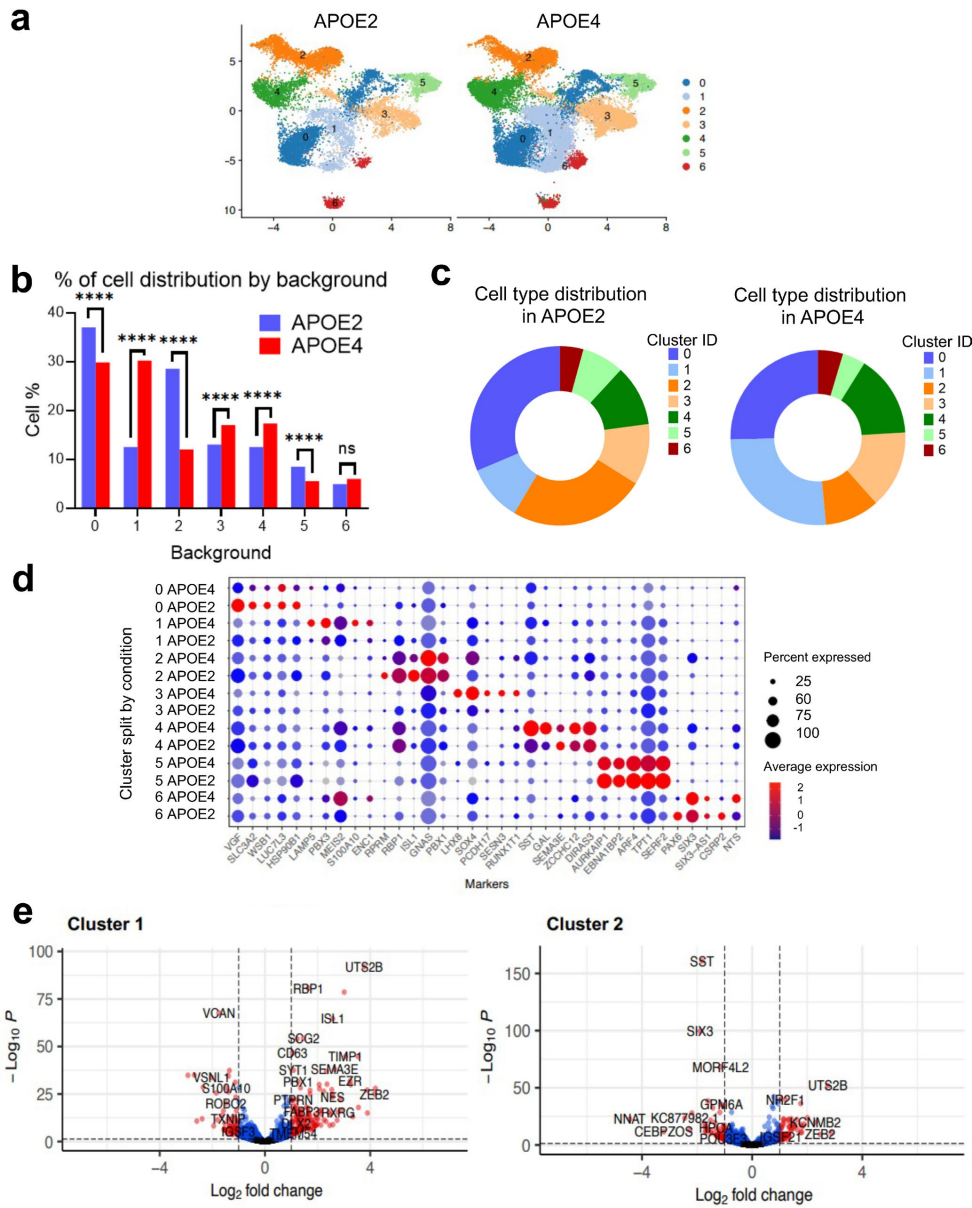
*APOE* genotypes exhibit distinct transcriptional subpopulations of GABAergic neurons revealed by scRNA‐seq. (a) UMAP visualization displaying seven transcriptionally distinct clusters derived from 7328 high‐quality GABAergic neurons from *APOE2* and *APOE4* genotypes. (b) Proportional distribution of *APOE2* and *APOE4* neurons across clusters, showing enrichment of *APOE4* cells in clusters 1, 3, and 4, and *APOE2* neurons in clusters 0, 2, and 5. Cluster 6 is evenly distributed. Percentage cell distribution per cluster is shown. ****p < 0.0001. (c) Pie charts showing cell type distribution within each genotype. (d) Cluster marker genes identifying the most representative genes for each neuronal cluster. (e, f) Volcano plots showing DEGs between *APOE2* and *APOE4* neurons within clusters 1 (*APOE4*‐enriched) and 2 (*APOE2*‐enriched).

To assess genotype‐specific transcriptional differences at single‐cell resolution, we performed 10x Genomics single‐cell RNA sequencing (scRNA‐seq) on GABAergic neurons derived from *APOE2* and *APOE4* backgrounds. After quality filtering (e.g., retaining cells with ≥ 300 features, > 1000 unique molecular identifiers (UMIs), and < 25% mitochondrial reads), transcriptomes from 7328 cells were retained for analysis. Using the Seurat analysis pipeline, we identified seven transcriptionally distinct clusters, with cells from both genotypes across all clusters (Figure 3a) (Satija et al. 2015).

Although both genotypes contributed to each cluster, their relative distributions differed. Clusters 1, 3, and 4 were enriched for *APOE4* neurons, whereas clusters 0, 2, and 5 were more enriched in the *APOE2* neurons (Figure 3b,c). Cluster‐specific marker genes were identified by differential expression analysis (Figure 3d) and visualized using violin plots (Figure S2a).

*APOE2* neurons have higher expression of VGF (nerve growth factor inducible) in Cluster 0 than *APOE4* neurons (Figure 3d). VGF is a neuropeptide precursor downregulated in AD (El Gaamouch et al. 2020), a top causal master regulator of AD networks (Beckmann et al. 2020), and overexpression of VGF rescues phenotypes in AD mice (El Gaamouch et al. 2020). Cluster1 (Figure 3e, Table S2, volcano plot E2/E4 comparison), enriched in *APOE4* neurons, has higher expression of *VCAN* (Versican), *TXNIP* (thioredoxin‐interacting protein), and *VSNL1* (Visinin‐like 1, aka VILIP‐1, HLP3). VSNL1 is a neuronal calcium sensor protein and is increased in the hippocampus and entorhinal cortex, which are affected early in AD (Groblewska et al. 2015; Halbgebauer et al. 2022; Tarawneh et al. 2015). Cluster 2, enriched for *APOE2* neurons, has high expression of neuronal genes *KCNMB2* (potassium calcium‐activated channel subfamily M regulatory beta) (Bentrop 2001) and *IGSF21* (immunoglobulin superfamily member 21). IGSF21 is expressed on the postsynaptic membrane and stabilizes inhibitory synapses (Tanabe et al. 2017).

To understand functional divergence among clusters, we performed gene set enrichment analysis (Figure S2b). Core “hallmarks” shared across clusters included TNF‐alpha signaling, hypoxia, and inflammatory pathways. Interestingly, cluster 0, enriched in *APOE2* neurons, showed specific enrichment for the mitotic spindle and in DNA damage response to UV light. Overall, pathway enrichment revealed a distribution of 24% inflammatory signaling and 19% DNA repair pathways (Figure S2c).

### 2.4 *APOE2* GABAergic Neurons Are Resistant to DNA Damage

**FIGURE 4:**
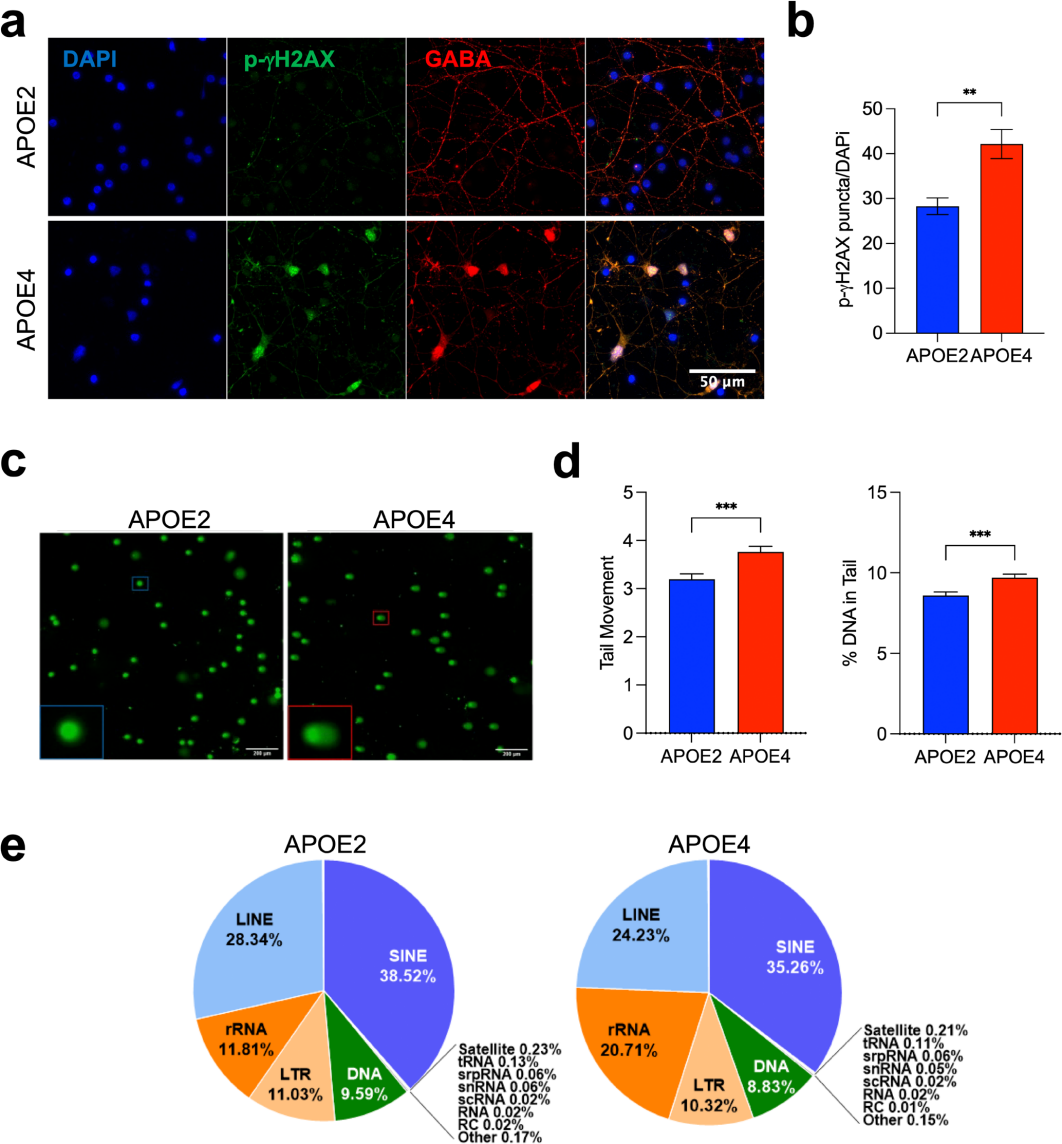
*APOE2* GABAergic neurons are resilient to DNA damage and exhibit altered rRNA repetitive element composition compared to *APOE4* neurons. (a) Representative immunocytochemistry images showing p‐γH2AX (green), a marker of DNA damage, GABA (red), and DAPI (blue). Scale bar: 50 μm. (b) Quantification of p‐γH2AX puncta in GABAergic neurons. Bars represent mean ± SEM; *n* = 3 biological samples, **p < 0.01. Scale bar, 50 μm. (c) Alkaline comet assay assessing the level of DNA damage in *APOE2* and *APOE4* neurons. Representative images showing the comet tail indicative of DNA strand breaks. (d) Quantification of Olive tail moment and percentage of tail DNA. Values represented are the combined values from three biological replicates, each containing > 1000 analyzed cells per genotype. Scale bar, 200 μm. (e) Pie charts showing the proportional composition of repetitive element classes in *APOE2* and *APOE4* neurons. The comparative analysis highlights genotype‐dependent differences in LTR, rRNA, and SINE elements within the repetitive element landscape.

Given the strong transcriptomic signature of *APOE2* GABAergic neurons enriched in DNA signaling, we assessed relevant phenotypes associated with DNA damage. Immunocytochemistry was performed on the isogenic *APOE2* and *APOE4* GABAergic neurons using the DNA‐damage repair‐associated protein p‐γH2AX (Figure 4a), which correlates with double‐stranded breaks and residual DNA damage (Ivashkevich et al. 2012; Paull et al. 2000; Sharma et al. 2012). p‐γH2AX staining was greater in *APOE4* than in *APOE2* GABAergic neurons (Figure 4b). To determine if the *APOE4* neurons undergo increased DNA damage rather than just altered repair pathways, we performed the comet assay to analyze DNA breaks (Larson 2016; Olive and Banath 2006) (Figure 4c). We examined the tail DNA percent (percentage of tail DNA over DNA of the entire cell) and the olive tail moment of the comets. The *APOE4* neurons had a greater percentage of DNA in their tails than *APOE2* neurons. The olive tail moment value parameter, a composite parameter reflecting DNA migration distance and distribution, was also greater for *APOE4* than for *APOE2* (Figure 4d). Thus, more DNA damage was found in *APOE4* than in *APOE2* GABAergic neurons.

Aberrant expression of repetitive elements (e.g., retrotransposons) is a hallmark of aging and neurodegenerative diseases (De Cecco et al. 2019; Saleh et al. 2019). These repetitive elements are often associated with hot spots of DNA damage (Argueso et al. 2008). To determine if the *APOE* genotypes of GABAergic neurons changed the expression of repetitive elements, we used RepEnrich2 to quantify the repetitive elements in our RNA‐seq data. We also used DESeq2 to identify differentially expressed repetitive elements (Table S3) (Love et al. 2014). PCA of the repetitive elements showed that *APOE2* and *APOE4* GABAergic neurons were clustered separately. We analyzed the composition of the repetitive element landscape, based on class membership, which includes repetitive rRNA (ribosomal RNA), LINE, SINE, snRNA, and LTR elements (Figure 4e). *APOE4* GABAergic neurons had greater rRNA expression and lower expression of SINE, LINE, and LTR elements (Figure 4e). Overall, our results suggest that *APOE2* neurons are more resistant to DNA damage and do not exhibit the increased rRNA expression observed in *APOE4* neurons. *APOE4* neurons have greater rRNA expression, which has been associated with cellular senescence and nucleolar stress (Morlot et al. 2019).

### 2.5 *APOE2* Ngn2‐Induced Glutamatergic Neurons Have Smaller Nucleoli

**FIGURE 5:**
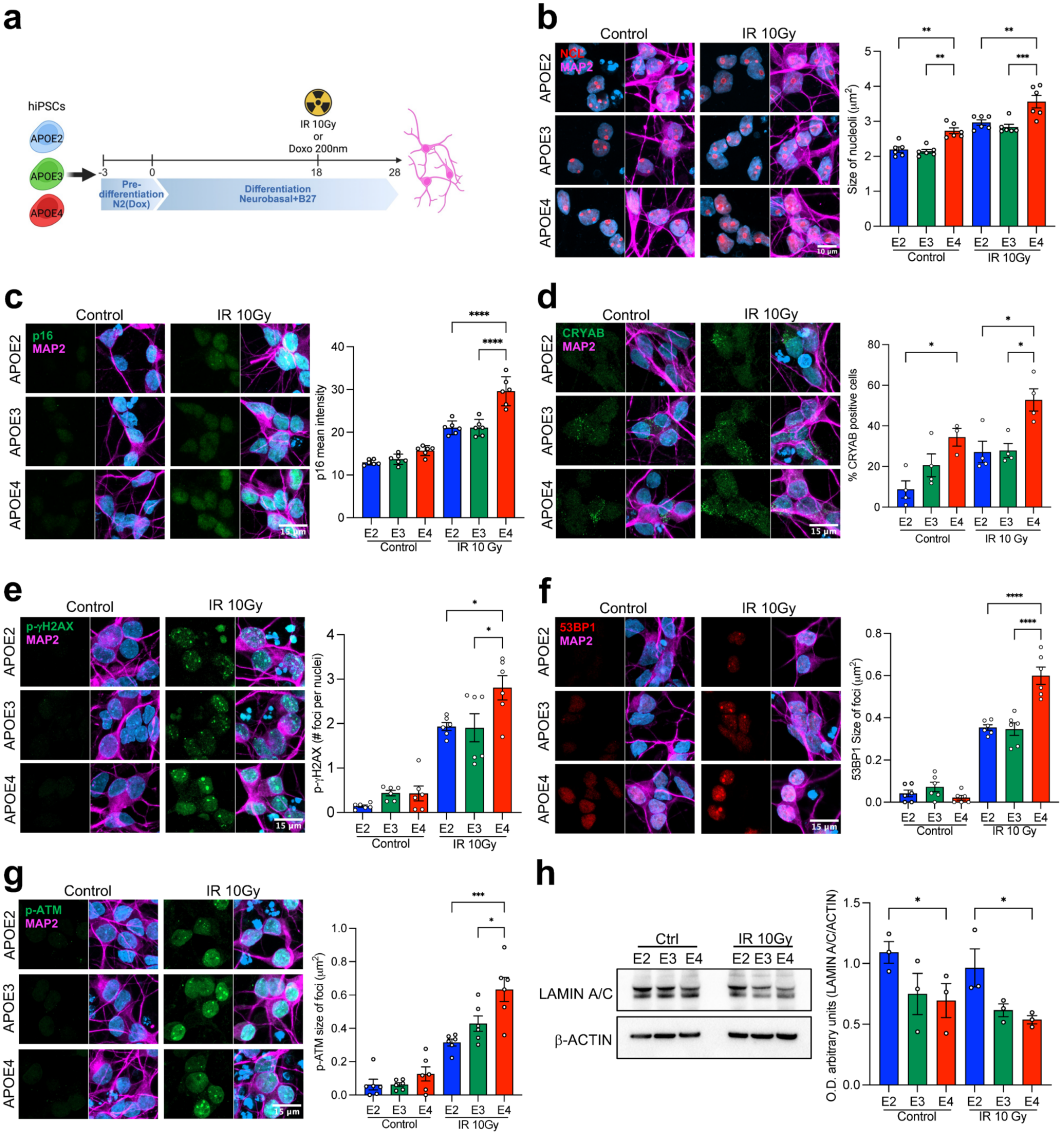
*APOE2* Ngn2‐induced glutamatergic neurons derived from human iPSCs exhibit features associated with longevity and are resilient to both irradiation‐induced senescence and DNA damage compared to *APOE4* neurons. (a) Schematic representation of the differentiation of human isogenic iPSCs into glutamatergic neurons via Ngn2 induction. The differentiation process spans 28 days. Ngn2 neurons were exposed to either irradiation (10 Gy) or doxorubicin (200 nM) at day 18 of differentiation to induce cellular senescence. (b) Representative immunocytochemistry images for Nucleolin (red), a marker of the nucleolus, MAP2 (violet), and DAPI (blue). Bar graphs represent the mean ± SEM showing nucleolar size. One‐way ANOVA followed by Tukey's post hoc test; *n* = 4 biological samples per genotype, **p < 0.01, ***p < 0.001. (c, d) Immunocytochemistry for senescence‐associated markers p16 and CRYAB in Ngn2 neurons of all three *APOE* genotypes. (c) Representative images showing p16 (green), MAP2 (violet), and DAPI (blue). Bar graphs represent the mean ± SEM showing p16 mean intensity. One‐way ANOVA, followed by Tukey's post hoc test; *n* = 4 biological samples per genotype, ****p < 0.0001. (d) Representative images showing CRYAB (green), MAP2 (violet), and DAPI (blue). Bar graphs represent the mean ± SEM showing the percentage of CRYAB‐positive cells. One‐way ANOVA followed by Tukey's post hoc test; *n* = 4 biological samples per genotype, *p < 0.05. (e) Immunocytochemistry for DNA damage marker p‐γH2AX in Ngn2 neurons. Representative images show p‐γH2AX (green), MAP2 (violet), and DAPI (blue). Bar graphs represent the mean ± SEM showing the number of p‐γH2AX foci per nucleus. One‐way ANOVA, followed by Tukey's post hoc test; *n* = 6 biological samples per genotype, *p < 0.05, ****p < 0.0001. (f, g) Immunocytochemistry for DNA damage response markers 53BP1 and p‐ATM in Ngn2 neurons. (f) Representative images showing 53BP1 (red), MAP2 (violet), and DAPI (blue). Bar graphs represent the mean ± SEM showing 53BP1 foci size. One‐way ANOVA followed by Tukey's post hoc test; *n* = 6 biological samples per genotype, ****p < 0.0001. (g) Representative images showing p‐ATM (green), MAP2 (violet), and DAPI (blue). Bar graphs represent the mean ± SEM showing p‐ATM foci size. One‐way ANOVA, followed by Tukey's post hoc test, *n* = 6 biological samples per genotype, *p < 0.05, ***p < 0.001. (h) Representative western blot showing LAMIN A/C levels across the *APOE* genotypes in Ngn2 neurons, with β‐ACTIN as a loading control. Bar graphs represent the mean ± SEM. One‐way ANOVA followed by Tukey's post hoc test; *n* = 3 biological samples per genotype, *p < 0.05.

*APOE2* GABAergic neurons show upregulation of categories related to DNA repair and stability, suggesting enhanced genomic maintenance capacity and relative resistance to DNA damage. Moreover, these neurons resist an increase in repetitive elements, another hallmark of aging associated with cellular senescence. Therefore, we evaluated whether *APOE2* might confer protection against cellular senescence and DNA damage induced by genotoxic stress using a second neuronal model—excitatory neurons—with all three human *APOE* genotypes. Using Cas9/CRISPR‐engineered isogenic male iPSCs with *APOE* alleles (*APOE* ε2/ε2, ε3/ε3, and ε4/ε4 genotypes), the iPSCs were differentiated into excitatory glutamatergic neurons using inducible expression of Neurogenin‐2 (Ngn‐2). After 28 days of differentiation, the cells expressed PSD95 (postsynaptic density protein‐95) and VGLUT1 (vesicular glutamate transporter 1), markers of mature glutamatergic neurons, as well as MAP2 (Microtubule‐associated protein 2), a pan neuronal marker expressed across all genotypes (Figure S3a). Neurons of all *APOE* genotypes expressed APOE as confirmed by immunocytochemistry and western blot analysis (Figure S3b,c). Subsequently, glutamatergic neurons were exposed to irradiation (10 Gy) or doxorubicin (200 nM) to evaluate key markers associated with DNA damage and cellular senescence (Figure 5a).

Long‐lived animals have decreased expression of rRNA and smaller nucleoli (Tiku et al. 2017), consistent with our previous observation of reduced rRNA levels in the *APOE2* GABAergic neurons. The nucleolus is a nuclear subcompartment for rRNA synthesis and ribosomal subunit assembly and plays a crucial role in genome integrity, nuclear architecture, stress signaling, and cell‐cycle regulation. Reduced nucleolar size has been associated with longevity and metabolic health (Tiku et al. 2017). Therefore, we evaluated the effect of APOE isoforms on the nucleolar size by immunostaining against Nucleolin (NCL), a protein involved in the synthesis of rRNA. Strikingly, *APOE2* and *APOE3* glutamatergic neurons displayed significantly smaller nucleoli than *APOE4* neurons under basal conditions and irradiation‐induced senescence (Figure 5b). Thus, the nucleolar size of *APOE2* glutamatergic neurons aligns with a cellular feature associated with longevity and metabolic health.

### 2.6 *APOE2* Ngn2‐Induced Glutamatergic Neurons Are Resilient to Cellular Senescence

Cellular senescence, an important process in aging, is characterized by the upregulation of p16/CDKN2A and p21/CDKN1A, DNA damage response, nuclear enlargement, chromatin reorganization, and the expression of pro‐inflammatory factors. The morphological alterations of the nucleus can serve as a predictor of senescence (Heckenbach et al. 2022). Therefore, we evaluated the nuclear size of the *APOE* glutamatergic neurons. Following irradiation‐induced senescence, the nuclear size of glutamatergic neurons increased in all genotypes compared to control conditions. The nuclear size of *APOE2* neurons was smaller than that of *APOE4* (Figure S4a). To determine whether APOE isoforms are differentially susceptible to genotoxicity‐induced cellular senescence, we measured p16 expression. Irradiation significantly induced p16 in all genotypes compared to the control conditions. Notably, *APOE4* neurons showed higher levels than *APOE2* and *APOE3* (Figure 5c).

We further assessed CRYAB (αB‐crystallin), a small heat shock protein, which is upregulated in senescent cells and can be used as a senolytic target (Limbad et al. 2022). Notably, *APOE2* neurons displayed a lower percentage of CRYAB‐positive cells than *APOE4* under basal conditions. Likewise, CRYAB‐positive cells increased after irradiation‐induced senescence in all genotypes, compared to controls, but *APOE4* neurons contained a higher proportion of CRYAB‐positive cells than *APOE2* and *APOE3*. Interestingly, more CRYAB‐positive cells were in *APOE4* neurons under basal conditions than in *APOE2* neurons (Figure 5d). Altogether, these results suggest that *APOE2* glutamatergic neurons are resilient to genotoxicity‐induced senescence and that *APOE4* neurons are more prone to acquire a senescent‐associated phenotype.

### 2.7 *APOE2* Ngn2‐Induced Glutamatergic Neurons Are Resistant to DNA Damage

As described above, *APOE2* GABAergic neurons exhibited upregulation of pathways related to DNA damage response and repair. We therefore evaluated whether *APOE2* confers protection against DNA damage and resistance to genotoxicity‐induced senescence following irradiation or doxorubicin treatment. DNA damage accumulates with age due to increased reactive oxygen species production and declining DNA repair, leading to DNA damage response and cellular senescence. Irradiation significantly increased p‐γH2AX, 53BP1, and p‐ATM across all genotypes, compared to control conditions, consistent with activation of DNA damage response, a key driver of senescence. Notably, *APOE4* glutamatergic neurons exhibited a higher number of p‐γH2AX foci per nucleus, as well as larger foci, compared to *APOE2* under irradiation‐induced senescence (Figure 5e). Similarly, 53BP1 and p‐ATM analysis revealed that following irradiation, *APOE4* neurons had increased foci size compared to *APOE2* and *APOE3* neurons (Figure 5f,g). The statistically significant change in p‐ATM foci size during irradiation was more pronounced in *APOE3* and *APOE4* neurons relative to *APOE2* neurons. Interestingly, the basal levels of 53BP1 mean intensity were *APOE* allele‐dependent (Figure S4b).

Correspondingly, in doxorubicin‐induced cellular senescence, *APOE2* neurons had lower levels of p16 than *APOE3* and *APOE4* neurons (Figure S5a). Doxorubicin significantly increased p‐γH2AX, 53BP1, and p‐ATM across all genotypes compared to control conditions. Moreover, *APOE4* neurons displayed greater p‐γH2AX numbers of foci per nucleus, larger 53BP1, and p‐ATM than *APOE3* and *APOE2* (Figures S5b and S6a,b). Collectively, these findings indicate *APOE2* glutamatergic neurons are resilient to DNA damage after genotoxic stress.

To evaluate early DNA damage response dynamics, we performed a time course analysis in *APOE* neural stem cells (NSCs). Consistent with the genotype‐specific response to DNA damage observed in differentiated neurons, p‐γH2AX, p‐ATM, and 53BP1 resolved more rapidly in *APOE2* NSCs compared to *APOE4* NSCs (Figure S7). These results suggest that APOE2 promotes a more efficient DNA damage response.

Given that DNA damage can lead to epigenomic alterations and nuclear instability, we examined the nuclear lamina integrity and heterochromatin status. Loss of nuclear lamina components and heterochromatin is associated with genomic instability and aberrant transcription during cellular senescence. Therefore, we evaluated LAMIN A/C, a major component of the nuclear lamina, as well as H3K9me3, a heterochromatin‐associated histone modification linked to chromatin organization and cellular senescence. Under basal conditions, *APOE3* and *APOE4* neurons displayed lower LAMIN A/C levels compared to *APOE2* neurons (Figure 5h). Notably, *APOE2* neurons were resistant to irradiation‐induced downregulation of LAMIN A/C observed in *APOE3* and *APOE4* neurons (Figure 5h). In contrast, *APOE4* neurons exhibited higher basal H3K9me3 levels compared to *APOE2* and *APOE3* neurons. Notably, H3K9me3 levels decreased in *APOE3* and *APOE4* neurons relative to control conditions but not in *APOE2* neurons (Figure S4c). These findings may suggest that *APOE2* glutamatergic neurons maintain greater nuclear and chromatin stability following genotoxic stress.

### 2.8 Recombinant APOE2 Confers Protection Against Irradiation‐Induced DNA Damage

**FIGURE 6:**
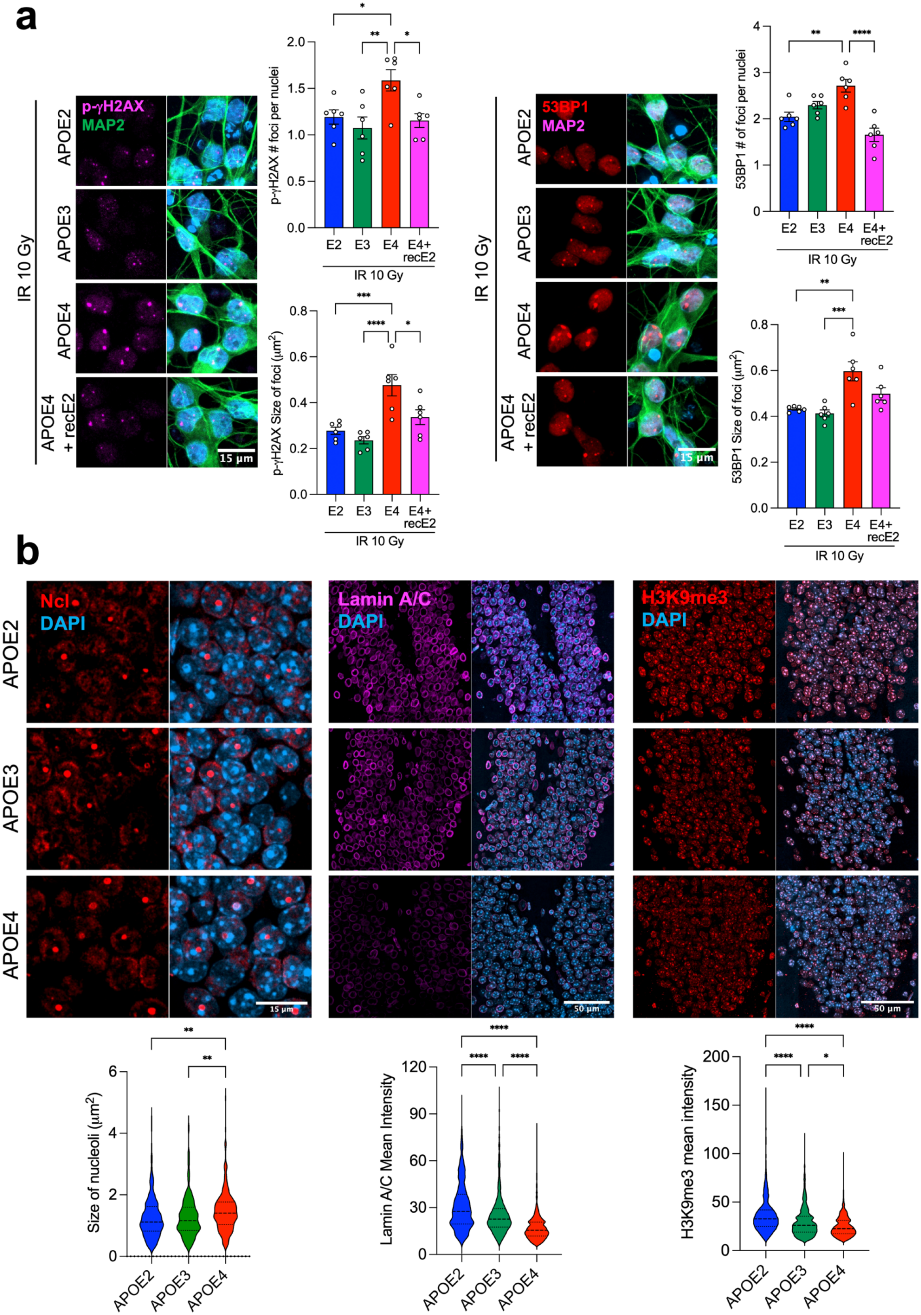
Recombinant APOE2 confers protection against irradiation‐induced DNA damage, and the hippocampus of aged *APOE2* knock‐in mice displays molecular features associated with longevity. (a) Immunocytochemistry for DNA damage and repair markers p‐γH2AX and 53BP1 in Ngn2 neurons of all three *APOE* genotypes following recombinant APOE2 treatment. Representative images show p‐γH2AX (violet), MAP2 (green), and DAPI (blue). Bar graphs represent the mean ± SEM showing p‐γH2AX and 53BP1 number of foci per nucleus and the size of foci. One‐way ANOVA followed by Tukey's post hoc test; *n* = 3, *p < 0.05, **p < 0.01, ***p < 0.001, ****p < 0.0001. (b) Immunohistochemistry for markers associated with nuclear homeostasis, including Nucleolin, Lamin A/C, and H3K9me3, in the hippocampus of aged *APOE* knock‐in mice. Representative images show Nucleolin (red) and DAPI (blue); Lamin A/C (violet) and DAPI (blue); and H3K9me3 (red) and DAPI (blue) across *APOE* genotypes. Violin plots show nucleolar size, Lamin A/C levels, and H3K9me3 levels. One‐way ANOVA followed by Tukey's post hoc test; *n* = 3–4 mice per genotype at 16 months of age, *p < 0.05, **p < 0.01, ***p < 0.001, ****p < 0.0001.

As described above, *APOE2* GABAergic neurons exhibited reduced DNA damage, and *APOE2* glutamatergic neurons were more resilient to genotoxic‐induced DNA damage. We therefore tested whether recombinant APOE2 treatment could protect *APOE4* glutamatergic neurons from irradiation‐induced DNA damage. Recombinant APOE2 treatment reduced p‐γH2AX and 53BP1 number of foci per nucleus and foci size following irradiation (Figure 6a). These results indicated that APOE2 attenuates irradiation‐induced DNA damage signaling in *APOE4* neurons.

### 2.9 Validation of Aging‐Associated Molecular Features in *APOE* Knock‐In Mice

Next, we evaluated age‐related markers in brain tissue from 16‐month‐old *APOE* knock‐in mice. We focused on the dentate gyrus, a region critically involved in learning and memory and vulnerable in AD. *APOE2* and *APOE3* knock‐in mice displayed smaller nucleoli compared to *APOE4* mice (Figure 6b), consistent with the association between nucleolar enlargement and aging. We next assessed the nuclear lamina integrity by measuring Lamin A/C levels. *APOE2* knock‐in mice had higher levels of Lamin A/C than *APOE3* and *APOE4* mice (Figure 6b), consistent with preserved nuclear structure. We also evaluated H3K9me3 levels, a heterochromatin‐associated histone modification linked to chromatin organization and aging. Aged *APOE4* and *APOE3* mice exhibited reduced levels of H3K9me3 in the dentate gyrus compared to *APOE2* mice (Figure 6b). In addition, we quantified nuclear Hmgb1 levels, as Hmgb1 translocation to the cytosol is associated with senescence. Aged *APOE2* mice retained higher nuclear Hmgb1 levels compared to *APOE3* and *APOE4* mice (Figure S8a). Finally, using publicly available RNA‐seq data from 18‐month‐old *APOE* mice, we performed enrichment analysis for the Hallmarks of Aging gene sets (Labuza et al. 2025). Gene sets related to cellular senescence were significantly enriched in *APOE4* compared to *APOE2* mice (Figure S8b). Collectively, these findings support the conclusion that APOE2 is associated with molecular features indicative of healthier aging in vivo.

## 3 Discussion

The *APOE* genotype is the strongest genetic modifier of Alzheimer's disease risk and is also associated with differences in lifespan; however, the molecular mechanisms underlying these divergent aging trajectories remain poorly understood. In this study, we demonstrate that *APOE* alleles differentially regulate neuronal genome maintenance and susceptibility to senescence. Across human isogenic iPSC‐derived GABAergic and glutamatergic neurons, APOE2 was associated with reduced endogenous DNA damage, enhanced transcriptional enrichment of DNA repair pathways, and more efficient resolution of genotoxic stress, whereas *APOE4* neurons exhibited persistent DNA damage signaling and elevated expression of rRNA repetitive elements. Consistent with these transcriptional and functional differences, *APOE2* neurons displayed smaller nucleoli and preserved nuclear lamina integrity—features linked to longevity and genomic stability—while *APOE4* neurons showed enlarged nucleoli and increased senescence‐associated markers. Importantly, these anti‐aging molecular features were recapitulated in the hippocampus of aged *APOE2* knock‐in mice, supporting the in vivo relevance of our findings. Together, these results suggest that *APOE* genotype shapes neuronal aging through differential regulation of DNA damage responses and nuclear homeostasis.

Evidence consistently links the *APOE4* allele to a wide range of pathological mechanisms in AD. This genotype has been associated with increased amyloid‐β aggregation, impaired amyloid‐β clearance, neuroinflammation, synaptic dysfunction, tau pathology, and mitochondrial impairment (Kim et al. 2009). Notably, in the context of AD, selective removal of neuronal APOE4 expression significantly reduced tau accumulation, gliosis, neurodegeneration, and myelin deficits (Koutsodendris et al. 2023). In contrast, only a few studies focused on the role of the APOE2 isoform in neurons. In primary hippocampal neurons, treatment with APOE2 or APOE3 protein increased the extent and complexity of the dendritic arbor and enhanced the frequency of mature spines (Diaz et al. 2022). In this study, we found that APOE2 increases neuronal outgrowth and mobility in GABAergic neurons.

Aging and neurodegenerative diseases are marked by genomic instability in neurons, including dysregulation of repetitive elements expression and activity (Guo et al. 2018). In senescent human mesenchymal progenitor cells, the accumulation of APOE drives increased expression of repetitive elements (Zhao et al. 2022). Additionally, rRNA production increases with age, leading to enhanced ribosome biogenesis, increased protein translation, and intracellular energy depletion (Buchwalter and Hetzer 2017). In our study, analysis of repetitive elements in GABAergic neurons revealed increased rRNA expression in *APOE4* genotype under non‐stress conditions, suggesting that *APOE* alleles differentially influence nucleolar activity.

The nucleolus is a subnuclear structure where rRNA is synthesized and assembled into ribosomal subunits. During aging, the nucleolus undergoes enlargement, a change linked to several detrimental mechanisms, including increased ribosome biogenesis, DNA damage, and genomic instability. In contrast, multiple lifespan‐extending interventions reduce nucleolar size across different species (Tiku et al. 2017). In this study, we observed reduced nucleolar size in *APOE2* and *APOE3* glutamatergic neurons under basal conditions. Conversely, *APOE4* neurons exhibited enlarged nucleoli, correlating with the dysregulation of rRNA expression in *APOE4* GABAergic neurons. These findings suggest that APOE plays a critical role in regulating genomic stability and consequently, nucleolar activity. In progeria syndrome and during cellular senescence, disruption of nuclear envelope components triggers global heterochromatin environment dysregulation, including repressive marks normally found on inactive rDNA (Buchwalter and Hetzer 2017; Freund et al. 2012). This, in turn, allows RNA polymerase I hyperactivity, resulting in nucleolar enlargement and enhanced ribosome biogenesis. Based on these insights, we hypothesized that the APOE2 isoform may contribute to the maintenance of nuclear envelope integrity, thereby preserving genomic stability and preventing nucleolar stress, whereas the APOE4 isoform may fail to maintain nuclear homeostasis. Future experiments are required to fully test this hypothesis and determine the precise molecular mechanism underlying this effect.

During aging, senescent cells gradually accumulate, contributing to tissue dysfunction and the onset of age‐related disorders, including AD (Lopez‐Otin et al. 2023). In senescent human mesenchymal progenitor cells, increased APOE expression disrupts nuclear envelope homeostasis and heterochromatin organization. This occurs through the degradation of nuclear lamina and heterochromatin‐associated proteins via autophagy (Zhao et al. 2022). However, the effects of *APOE* alleles on neuronal senescence remain unknown. Here we demonstrate that the APOE2‐expressing glutamatergic neurons are resistant to stress‐induced cellular senescence, showing lower levels of p16 and CRYAB than *APOE4* neurons. Moreover, under basal conditions, *APOE4* glutamatergic neurons showed higher levels of p16 and CRYAB, suggesting that they are prone to acquire the senescent phenotype. Therefore, these findings reveal a novel mechanism by which APOE2 might promote exceptional longevity and protection against AD.

DNA damage affects all aspects of the aging phenotype and is recognized as a hallmark of cellular senescence (d'Adda di Fagagna 2008; Schumacher et al. 2021). It leads to various molecular consequences, including genome instability, telomere dysfunction, epigenetic alterations, among others (Schumacher et al. 2021). In AD, DNA damage and, particularly, double‐strand breaks, actively contribute to disease pathogenesis. Although previous studies have reported no significant differences in DNA damage between *APOE4* carriers and noncarriers, the APOE4 isoform has been associated with mitochondrial dysfunction and increased oxidative stress, which may ultimately promote DNA damage (Blumenfeld et al. 2024; Chua et al. 2015). In our study, we found that under basal conditions, *APOE2* GABAergic neurons display reduced DNA damage, as measured by comet assay and p‐γH2AX immunostaining. Furthermore, the transcriptomic analysis revealed enrichment of pathways related to DNA repair. In response to stress‐induced senescence, *APOE2* glutamatergic neurons also accumulated less DNA damage, compared to *APOE4*. Additionally, recombinant APOE2 treatment attenuated irradiation‐induced DNA damage in *APOE4* neurons. However, the precise mechanism by which APOE2 confers protection against double‐strand breaks remains to be elucidated.

In response to DNA damage, cells activate a mechanism known as DNA‐damage response (DDR), which detects DNA lesions, signals their presence, and promotes their repair (Jackson and Bartek 2009). Defects in the DDR contribute to AD pathogenesis, leading to increased DNA damage and neurodegeneration (Lin et al. 2020; Nelson and Xu 2023). Moreover, a persistent DDR can be detrimental and promote cellular senescence (Fumagalli et al. 2012). We evaluated the levels of 53BP1 and p‐ATM, two key components of the DDR, under conditions of stress‐induced senescence. Our results revealed a lower number and smaller 53BP1 and p‐ATM foci in *APOE2* glutamatergic neurons after irradiation, whereas *APOE4* neurons exhibited a higher number and larger foci. DDR can influence genomic instability through the degradation of heterochromatin remodelers, such as EZH2. Upon DNA damage, activation of p‐ATM leads to EZH2 degradation, resulting in loss of heterochromatin, increased DNA damage, and the onset of cellular senescence (Ito et al. 2018). Thus, in *APOE4* neurons, unresolved DNA damage and persistent activation of the DDR likely contribute to the induction of cellular senescence.

Nuclear lamina proteins, including LAMIN B1 and LAMIN A/C, levels decline with normal aging, contributing to nuclear instability, disruption of nuclear pore complex organization, and accumulation of DNA damage. Loss of LAMIN B1 is a feature of cellular senescence and is associated with altered nuclear morphology and widespread changes in gene expression (Freund et al. 2012; Matias et al. 2022). Mutations in the *LMNA* gene cause Hutchinson‐Gilford Progeria Syndrome (HGPS), resulting in LAMIN A/C deficiency and accelerated aging (Gonzalo et al. 2017). Our results showed that *APOE2* neurons had resilience to loss of LAMIN A/C levels after irradiation. Likewise, the hippocampus of old *APOE2* knock‐in mice showed higher levels of Lamin A/C. Previous findings revealed that increased APOE leads to nuclear lamina protein degradation and subsequently heterochromatin destabilization (Zhao et al. 2022). LMNA depletion promotes replicative stress and accumulation of DNA damage (Schultz et al. 2025). Therefore, we hypothesized that APOE2 plays a role in stabilizing LAMIN A/C, which in turn prevents genomic instability. Further experiments are required to define the molecular mechanisms underlying this effect.

Collectively, our findings support a model in which the *APOE* genotype influences neuronal aging trajectories through regulation of genomic stability. APOE2 is associated with enhanced DNA repair capacity, preserved nuclear architecture, and restrained nucleolar activity, whereas APOE4 is linked to persistent DNA damage signaling, chromatin instability, and acquisition of senescence‐associated features. This genotype‐dependent divergence may contribute to differential vulnerability to neurodegeneration during aging. These protective mechanisms highlight a unique role for APOE2 in promoting healthy neuronal aging, while offering resistance to AD pathology. These insights open new avenues for the development of therapeutic strategies aimed at targeting DNA repair pathways and senescence to mitigate aging and neurodegeneration.

## 4 Materials and Methods

### 4.1 Culturing of *APOE2* and *APOE4* GABAergic Neurons

Human *APOE4* GABAergic neurons (Fujifilm, R1168, 4 million cells per vial) and *APOE2* GABAergic neurons (Fujifilm, R1169, 4 million cells per vial) were obtained from Fujifilm (formerly Cellular Dynamics International). Cells were thawed and maintained according to the manufacturer's instructions using iCell Neural Base Media 1 and its associated Supplement A (R1150), which together constitute the Complete Maintenance Medium. For culture, 8‐well chamber slides, 12‐well plates, 6‐well plates, and/or 96‐well plates were coated with one of two matrix conditions. The first condition, plates were coated with 0.01% poly‐L‐ornithine (Sigma‐Aldrich, P4957) and left overnight at 37°C, rinsed 3× each with Milli‐Q water (Elga Superflex Model PF3), and then coated with 3.3 μg/mL laminin (Sigma‐Aldrich, L2020) in Milli‐Q water for 1 h at 37°C. Wells were subsequently filled with Complete Maintenance Media. In the second condition, plates were coated with 0.1 mg/mL poly‐D‐lysine (Sigma‐Aldrich, P6407) overnight at 37°C, rinsed three times with Milli‐Q water, and then coated with Matrigel (130–160 μg/mL, Corning, CB40234) overnight at 37°C. Wells were aspirated and replenished with SynaptoJuice B as described in (Kemp et al. 2016). For most experiments, SynaptoJuice B was used, as the GABAergic neurons exhibited improved viability and morphology in this medium. Media were changed every 3–5 days. The cells were allowed to mature for at least 14 days in culture, and 2–5 batches were used depending upon the experiment.

### 4.2 Neuronal Network Analysis

*APOE4* and *APOE2* GABAergic inhibitory neurons were cultured as described above and imaged in brightfield every 3 h using a BioTek Cytation 5 instrument at 4× or 10× magnification. Image acquisition and analysis were performed using Gen5 software (BioTek, Version 3.08). Images were preprocessed using background correction and deconvolution (3× iterative cleaning step) to reduce optical blur and enhance neurite visibility. Processed images were used for neurite outgrowth quantification and for the generation of time‐lapse videos for cell motility analysis. Neurite outgrowth was quantified by applying a primary mask to processed images, with a mask threshold of 750 units. The “Sum Perimeter” (SP) parameter, defined as the total perimeter of all detected objects within the field of view, was extracted for each image. Objects within an acceptable size range of 5–500 μm were included in the analysis. Once optimized, mask parameters were applied uniformly across all wells and time points. To normalize for baseline variability, SP values at each time point were divided by the SP value of the first time point (read 1) for the corresponding well, generating a normalized Sum Perimeter (SP_norm). Temporal changes in SP_norm were plotted using GraphPad Prism 8.

### 4.3 Cell Movement Analysis

GABAergic neurons were cultured and imaged at 4× magnification as described above. For each well, 2 × 2 tiled images centered within the well were acquired and stitched using BioTek software. Time‐lapse image sequences were registered and exported as H.264‐compressed (MP4) videos. Cell body motility was analyzed using Image Analyst MKII (Image Analyst Software, Novato, CA). Cell bodies were identified in brightfield images using band‐pass spatial filtering with absolute value transformation, followed by image segmentation based on a modified version of the “Measure tracking parameters of cells in brightfield time lapses” pipeline. Individual cells were tracked across frames using a simulated annealing optimization algorithm implemented in the “Track Objects” function, which associates objects between consecutive frames based on proximity, size, and shape features without requiring direct spatial overlap. Tracks shorter than 10 frames were excluded from analysis. Because cell movement in culture is largely random, the vector sum of all displacement vectors within a field of view was assumed to be zero; any residual displacement was attributed to imperfect frame registration and was subtracted from each frame‐to‐frame displacement vector. Erroneous tracking events were excluded using a velocity threshold cutoff. The remaining tracks were quantified using the “Plot Tracking Parameters” function. Mean curvilinear velocity (VCL) was calculated as the average absolute velocity along the entire track. Average path velocity (VAP) was determined by smoothing velocity vectors prior to calculating absolute velocity, thereby reducing values for trajectories with frequent directional changes while preserving velocities of straight‐moving cells. Wobble was calculated as: $\text{Wobble}=1-\frac{\mathrm{VAP}}{\mathrm{VCL}}$. A value of 0 indicates smooth, linear motion, whereas higher values reflect increased directional variability. Tracking parameters were calculated for each cell and subsequently averaged across fields and wells.

### 4.4 Immunocytochemistry of *APOE2* and *APOE4* GABAergic Neurons

GABAergic inhibitory neurons were cultured for 6–10 days on 8‐well chamber‐slides (BD Falcon, 354108), fixed with 4% paraformaldehyde (Sigma, 158127) for 15 min at RT and washed 3× with PBS. Neurons were permeabilized with 0.1% Triton X‐100 (Fisher Scientific, BP151‐100) in PBS solution for 15 min, washed with PBS, and then blocked 30–60 min with 1% BSA (Sigma Aldrich, 03117332001) and 5% donkey serum (Sigma Aldrich, D9663) in PBS. An additional PBS wash was performed, and the cells were probed with primary antibodies overnight at 4°C. Neurons were washed 3× with PBS, and then probed with antigen‐matched, fluorescent‐labeled secondary antibodies for at least 1.5 h and washed 3× times with PBS in the dark. The cells were mounted with 12‐mm glass coverslips (48393‐251) using Prolong Gold with DAPI (ThermoFisher, P36931). Imaging was done using a Zeiss LSM 780 confocal on an Axio Observer Z1 inverted microscope with Plan‐Apochromat 63×/1.40 NA Oil DIC objective. Primary antibodies (1:100 dilution) include: VGAT (Synaptic Systems, 131011), GluR1 (Millipore, ABN241), PSD95 (Sigma, SAB5600103), GAD (Millipore, AB1511), phospho‐γH2AX (Millipore, 05‐636), Nestin (Abcam, ab92391), GABA (Synaptic Systems, A2052), MAP2 (Abcam, AB5622), P16 (Abcam, AB108349), Calbindin (Abcam, AB108404), Nucleolin (Abcam, AB22758), phospho‐ATM (ThermoFisher Scientific, MA1‐2020), 53BP1 (Cell Signaling, 4937), and H3K9me3 (Abcam, AB8898). Secondary antibodies (1:200–1:350 dilution) include: donkey‐anti mouse Alexa Fluor 488 (ThermoFisher, A21202) and donkey‐anti rabbit Alexa Fluor 555 (ThermoFisher, A32794). The phospho‐γH2AX images were analyzed using the Cytation 5 Biotek system quantifying the puncta in *APOE4* and *APOE2* GABAergic neurons.

### 4.5 RNA Extraction of *APOE2* and *APOE4* GABAergic Neurons and RNA Sequencing

Bulk RNA was extracted from 12‐well plates using the Bioline Isolate II RNA mini kit (Bioline, BIO‐52073) and the manufacturer's protocol. Each well contained approximately 400,000 GABAergic inhibitory neurons. The resulting elute was quantified on the NANODROP 2000 (D632) and frozen at −20°C. Three samples of each genotype for six total samples were sequenced by the UC‐Davis core. FASTQ files were assessed for sequencing quality using per‐base quality and adapter content. Reads were trimmed with Trimmomatic to remove adapter sequences and low‐quality bases. Trimmed reads were aligned to the human reference genome, and gene counts were generated by assigning aligned reads to annotated genes. Differential gene expression was performed using DESeq2, and low‐expressed genes were filtered out before testing. *p* values were corrected for multiple testing using the Benjamini‐Hochberg false discovery rate (FDR).

### 4.6 Gene Set Enrichment Analysis

GO was performed using a ranked list of differential gene expression with parameters set to 2000 gene‐set permutations and gene‐set size between 15 and 200. The gene‐sets included for the Gene Set Enrichment analyses were obtained from Gene Ontology (GO) database (GOBP_AllPathways), updated September 01, 2019 (http://download.baderlab.org/EM_Genesets/). An enrichment map (version 3.2.1 of Enrichment Map software (Merico et al. 2010)) was generated using Cytoscape 3.7.2 using significantly enriched gene‐sets with an FDR < 0.05. Similarity between gene‐sets was filtered by Jaccard plus overlap combined coefficient (0.375). The resulting enrichment map was further annotated using the AutoAnnotate Cytoscape App.

### 4.7 Single‐Cell RNA Isolation and Analysis of *APOE2* and *APOE4* GABAergic Neurons

Wells from 12‐well plates were harvested for use in single‐cell analysis. Three wells per genotype were harvested, for a total of six samples. We utilized the V3 Chromium Single Cell 3′ Reagent Kits User Guide and its associated Chromium i7 Multiplex Kit (10X, PN‐120262). Data have been deposited in NCBI's Gene Expression Omnibus GEO Series accession number GSE143276.

### 4.8 Single‐Cell RNAseq Analysis

FASTQ files from 10× single‐cell RNA sequencing were processed using Cell Ranger (GRCh38, 2020). Downstream bioinformatics analysis was performed in R using Seurat v4.3.0. Genes detected in fewer than three cells were removed. To filter out cells with low‐complexity libraries, the following thresholds were applied: number of features ≥ 300, log10(GenesPerUMI) > 0.85, mitochondrial RNA < 25%, and ribosomal RNA > 5%. Doublets were identified using DoubletFinder v2.0.6, accounting for 4% of the dataset, and subsequently removed. The novelty score, defined as the ratio of the number of genes to UMIs, was also calculated to ensure that all retained cells exhibited high transcriptomic diversity (novelty score > 0.8). Counts were normalized and scaled with *SCTransform*. Samples were batch‐corrected using the Harmony method. Unsupervised clustering based on transcriptional data was performed using Seurat's *FindNeighbors* and *FindClusters* functions. Clusters were visualized with *RunUMAP* and *DimPlot* using default settings. To characterize clusters, we used *FindConservedMarkers* and *FindAllMarkers*. Differential expression analysis was performed using a pseudo‐bulk approach and DESeq2 v1.42.1. Gene set and pathway enrichment analyses were conducted using clusterProfiler v4.10.1 and fgsea v1.28.0.

### 4.9 *APOE2* and *APOE4* GABAergic Inhibitory Neurons Comet Assay

Female homozygous *APOE2* (Fujifilm, R1169 donor ID 01434), and *APOE4* (Fujifilm, R1168, donor ID 01434) iCell GABAergic neurons were cultured in Corning 6‐well plates (Thermo Fisher Scientific, 140675) coated with 0.1 mg/mL poly‐D‐lysine (Sigma‐Aldrich, P6407) overnight at 37°C. Wells were rinsed 3× with Milli‐Q water and subsequently coated with 130–160 μg/mL Matrigel (Corning, CB40234) overnight at 37°C. SynaptoJuice B medium was added as previously described (Kemp et al. 2016). DNA damage was assessed using the alkaline comet assay (Trevigen/R&D Systems, 4250‐050‐03) according to the manufacturer's protocol. Approximately 3000 cells per condition were collected into 1.5‐mL microcentrifuge tubes (Sorenson, 11590) and mixed with 75 μL of pre‐warmed 1% low‐melting‐point agarose (Bio‐Rad, 1613102). The agarose–cell suspension was evenly spread onto Trevigen 2‐well comet slides and allowed to gel at 4°C for up to 30 min in the dark. Slides were immersed in pre‐chilled lysis solution (Trevigen, 4250‐050‐01) and incubated overnight at 4°C in the dark. For DNA unwinding, slides were transferred to freshly prepared alkaline unwinding solution (8 g NaOH and 2 mL 500 mM in 1 L distilled water; pH ≥ 13) and incubated for 1 h at 4°C in the dark. Slides were then placed in a Trevigen electrophoresis chamber (4250‐050‐ES) containing unwinding solution and subjected to electrophoresis at 21 V for 10 min at 4°C. Following electrophoresis, slides were washed twice with Milli‐Q water for 5 min each, rinsed once with 70% ethanol for 5 min, and air‐dried at 37°C in a carbon dioxide–free incubator for 10 min. DNA was stained with 1× SYBR Gold (Thermo Fisher Scientific, S11494) in Milli‐Q water for 30 min at room temperature in the dark. Slides were rinsed with Milli‐Q water and allowed to dry overnight at room temperature. Comets were imaged using a BioTek Cytation 5 imaging system (Gen5 software, version 3.08) and analyzed as described above.

### 4.10 Repetitive Repeat Element Analysis

We used RepEnrich2 to estimate the number of repetitive elements observed in our RNAseq data (Criscione et al. 2014). We used the repetitive element annotation for *Homo sapiens* from Repeatmasker.org. Once repetitive elements were quantified, DESeq2 was used to perform differential enrichment analysis (Love et al. 2014).

### 4.11 Generation of Isogenic Homozygote *APOE2* , *APOE3* , and *APOE4* iPSCs Using CRISPR/Cas9 Editing

The isogenic homozygote *APOE2*, *APOE3*, and *APOE4* iPSC lines were CRISPR edited using NCRM1 parental clones with Cas9 and gRNA (Synthego, Redwood City, CA). The three lines were fully sequenced. The homozygote lines had the expected sequences and no off‐target edits, and were karyotyped as normal. Human iPSC‐derived neurons were engineered to have a safe harbor locus with integration of a doxycycline‐inducible *Ngn2* transgene into *APOE2*, *APOE3*, and *APOE4* carrying human iPSCs (Wang et al. 2017). The TALENS and donor construct as 1:2 ratio. TALEN AAVS1A (3 μg), AAVS1B (3 μg), and pUC‐h*Ngn2*/oligo (6 μg) with puromycin selection were as described (Wang et al. 2017). All NCRM1 *APOE2*, *APOE3*, and *APOE4* PCR‐identified clones were karyotyped (Cell Line Genetics) and had normal karyotypes.

### 4.12 Differentiation of iPSCs Into Ngn2 Glutamatergic Neurons

We differentiated glutamatergic neurons from human isogenic iPSCs (*APOE* ε2/ε2, ε3/ε3, and ε4/ε4 genotypes) by inducible *Ngn2* expression, using a transgenic cassette that was integrated into the AAVS1 safe harbor locus of each *APOE* iPSC clone as described (Wang et al. 2017). iPSCs were maintained in colonies in 6‐cm plates pre‐coated with Matrigel (Corning, 354234) using mTESR Plus basal medium (Stemcell, 100‐0274). For differentiation, iPSCs were detached into single cells with StemPro Accutase (Gibco, A1110501) and plated on a Matrigel‐coated plate at a density of 2.5 × 10^5^ cells/cm^2^. iPSCs were maintained using N2+ medium containing KO DMEM/F12 (ThermoFisher, 12660012), 1% N2 supplement (ThermoFisher, 17502001), 1× NEAA (ThermoFisher, 11140050), 10 ng/mL BDNF (Peprotech, 450‐02), 10 ng/mL NT3 (Peprotech, 450‐03), and 2 μg/mL doxycycline. The medium was changed every day. After 3 days of pre‐differentiation, cells were detached using StemPro Accutase and plated in poly‐D‐lysine (20 μg/mL) coated coverslips or plates at a density of 7.5 × 10^4^ cells/cm^2^ and maintained using NBA+ medium containing Neurobasal A (ThermoFisher, 108888022), 2% B27 (ThermoFisher, 17504044), 1x GlutaMAX (ThermoFisher, 35050061), 10 ng/mL BDNF (Peprotech, 450‐02), 10 ng/mL NT3 (Peprotech, 450‐03). Neurons were differentiated for 28 days, and half of the medium was changed every week. A subset of plates were exposed to irradiation (10 Gy) or doxorubicin treatment (200 nM) at day 18 during the differentiation and analyzed 10 days after treatment. For recombinant APOE2 experiments, a subset of plates was treated with recombinant APOE2 (50 ng/mL) starting at day 11 twice a week until analysis.

### 4.13 Immunocytochemistry of Ngn2 Glutamatergic Neurons

Differentiated neurons were cultured on coverslips, fixed with paraformaldehyde 4% for 15 min at RT, and stored in PBS 1× at 4°C. Neurons were blocked and permeabilized using 1X PBS, 5% fish gelatin (Sigma, G7041), and 0.5% Triton‐X‐100 (Sigma, X100) for 2 h at RT. Primary antibodies were incubated overnight at 4°C in blocking solution at a concentration of 1:250 for p‐γH2AX (Millipore, 07‐627), p‐ATM (ThermoFisher, MA1‐2020), 53BP1 (CellSignaling, 4937), CRYAB (Abcam, ab13496), H3K9me3 (Abcam, ab8898), 1:750 for p16/cdkn2a (Abcam, ab108349), and MAP2 (Novus Biologicals, NB300‐213). Secondary antibodies were incubated for 2 h at RT in blocking solution at a concentration of 1:500 for anti‐mouse Alexa 488 (ThermoFisher, A32766), anti‐rabbit Alexa 555 (ThermoFisher, A32794), and anti‐chicken Alexa 647 (ThermoFisher, A21443), followed by incubation with DAPI (1 μg/mL) for 10 min at RT. Coverslips were mounted on a slide using ProLong Gold antifade mountant and stored at 4°C in the dark. Images were taken using a confocal microscope ZEISS LSM 980. Images were analyzed using Fiji Image J.

### 4.14 *APOE* Neural Stem Cells Differentiation

Neural stem cells were differentiated from human isogenic iPSCs expressing *APOE2*, *APOE3*, or *APOE4* as previously described (Galicia Aguirre et al. 2025). Briefly, iPSCs were cultured in mTESR1 medium (STEMCELL Technology, 05850). Differentiation was initiated by inhibiting SMAD signaling with SB431542 (10 μM, Tocris, 1614) and LDN‐193189 (1 μM, Tocris, 6053) in mTESR1 medium. By day 10, the harvested aggregates were plated at low density on Matrigel‐coated (1 mL, 50 μg) 10‐cm dishes in N2B27 medium. This medium consisted of DMEM/F12 (Gibco, Thermo Fisher Scientific, 11320‐033) supplemented with 1× N2 (Thermo Fisher Scientific, 17502001), 1× B27 (Thermo Fisher Scientific, 17504001), 1× GlutaMAX (Thermo Fisher Scientific, 35050061), 1× Non‐Essential Amino Acids (Thermo Fisher Scientific, 11140050), β‐fibroblast growth factor (FGF, 25 ng/mL, PeproTech, 100‐18B), and penicillin/streptomycin (P/S, 100 U/mL, Thermo Fisher Scientific, 15140122). At day 14, neural rosettes were seeded into a Matrigel‐coated P12‐well plate in Neural Proliferation Medium. This medium contained Neurobasal (Thermo Fisher Scientific, 21103049), 1× B27 supplement (Thermo Fisher Scientific, 17504001), 1× GlutaMAX (Thermo Fisher Scientific, 35050061), leukemia inhibitory factor (10 ng/mL, PeproTech, 300‐05), and 100 U/mL P/S, supplemented with 25 ng/mL β‐FGF. NSCs derived from this process were passaged upon reaching confluency. For time‐course experiments, NSCs were plated in 96‐well plates coated with Matrigel at a density of 6.2 × 10^3^ cell/cm^2^, after 2 days, cells were exposed to irradiation 5Gy and fixed using 4% PFA.

### 4.15 Mouse Tissue

Female mice homozygous for human *APOE2*, *APOE3*, and *APOE4* genes, through targeted replacement of the mouse Apoe gene, were purchased from Taconic Biosciences (Rensselaer, NY) (catalog numbers, 1547‐F or ‐M, 1548F or ‐M,1549‐F or ‐M, B6.129P2‐Apoe<tm1 (APOE*2)Mae>N9, B6.129P2‐Apoe<tm1 (APOE*3)Mae>N9, B6.129P2‐Apoe<tm1(APOE*4) Mae>N9). Mice were generated on the C57BL/6 genetic background, with the mouse APOE gene replaced with the human *APOE* gene under the control of murine *APOE* regulatory sequences. The Buck Institute for Research on Aging animal facility is an AAALAC International‐accredited institution (Unit Number 001070). All protocols and procedures described herein were approved by the Buck's Institutional Animal Care and Use Committee.

### 4.16 Immunohistochemistry of Human *APOE* Knock‐In Mice Brain Tissue

Paraffin‐embedded brain tissue sections (2 μm thickness) were deparaffinized using a standard xylene‐based protocol and rehydrated through graded ethanol. Antigen retrieval was performed in Tris–EDTA buffer (10 mM Tris base, 1 mM EDTA, pH 9.0), followed by brief rinsing in PBS. Nonspecific binding was blocked by incubating sections in blocking buffer containing PBS supplemented with 10% normal donkey serum, 5% bovine serum albumin (BSA), and 0.3% Triton X‐100 for 2 h at room temperature in a humidified chamber. Sections were then incubated overnight at 4°C with primary antibodies diluted in blocking buffer. After washing with PBS, sections were incubated with appropriate cross‐adsorbed fluorophore‐conjugated secondary antibodies diluted in blocking buffer. To reduce tissue autofluorescence, sections were treated with TrueBlack (Biotium) for 1 min, followed by three washes in PBS. Slides were mounted using ProLong Gold Antifade Mountant containing DAPI (Thermo Fisher Scientific). Images were acquired using a Zeiss LSM 980 confocal microscope. Image analysis was performed using Cellpose and Fiji (ImageJ).

### 4.17 Statistics

Graphs and statistical analysis were performed using Graphpad Prism 10. For GABAergic neurons, we performed 3 independent experiments using unpaired Student's *t*‐test comparing *APOE2* versus *APOE4*. For Ngn2 glutamatergic analysis, we performed 3 to 6 independent differentiations with technical replicates, then we performed a one‐way or two‐way ANOVA, followed by Tukey's post hoc test, which applies the family‐wise error rate (FWER). All significant values are represented by asterisks showing adjusted *p* values displayed in this paper as p.

## Author Contributions

Conceptualization: C.G.‐O., S.M.S., and L.M.E.; Methodology: C.G.‐O., S.M.S., C.G.A., G.V‐.H., D.G., K.S., L.W., E.P., N.M., K.A.W., N.T.M., S.S., K.S., T.E.T., A.A.G., and E.P.; Investigation: C.G.‐O., S.M.S., C.G.A., N.M., J.S., and L.M.E.; Funding acquisition: C.G.‐O. and L.M.E.; Supervision: E.V., J.C., D.F., S.D.M., S.M., and L.M.E.; Project administration: L.M.E.; Writing‐original draft: C.G.‐O., S.M.S., K.A.W., and L.M.E.; Writing‐review and editing: C.G.‐O. and L.M.E. S.M. did this work before he joined the NIH.

## Funding

This work was supported by the National Institute on Aging (1RO1AG061879, 5P01AG066591, and T32 AG000266); Paul F. Glenn Center for Biology of Aging; CatalystX award from Alex and Bob Griswold. This work was in part funded by a grant from the Hevolution Foundation (HF‐PART‐23‐1422047).

## Conflicts of Interest

The authors declare no conflicts of interest.

## Supporting information


**Figure S1:** APOE expression and altered motility of GABAergic neurons. (a) Time course of label‐free bright field micrographs of GABAergic neurons recorded and analyzed to quantify cell motility for *APOE2* and *APOE4* genotypes. Representative movement tracks are shown. Each colored track represents the trajectory of a single identified cell body over a 112‐frame recording, including only cells tracked for at least 10 consecutive frames. Images were taken under brightfield. (b) Differential expression of *APOE* in GABAergic neurons. Bulk RNA‐seq raw counts of *APOE* transcripts. Bar plots represent mean ± SEM; t‐test, **p < 0.001, *n* = 3.
**Figure S2:** Enrichment analysis of *APOE* GABAergic neurons. (a) Most representative genes in each cluster detected in the single‐cell analysis. (b) Gene Set Enrichment Analysis (GSEA) of Hallmark pathways enriched across the seven clusters identified in *APOE* GABAergic neurons. Hallmark pathways include TNF‐α signaling, hypoxia, and inflammatory pathways across clusters. Cluster 0 was specifically enriched for mitotic spindle and UV‐induced DNA damage response pathways. (c) Pie chart summarizing pathway distribution across clusters: 24% inflammatory signaling,19% DNA repair, and 57% other pathways.
**Figure S3:** Ngn2 neurons derived from human isogenic iPSCs express markers of fully differentiated glutamatergic neurons across the three *APOE* genotypes. (a) Representative immunocytochemistry for PSD95 (red), VGLUT (green), MAP2 (violet), and DAPI (blue) with the genotypes indicated. (b) APOE expression in glutamatergic neurons. Representative images of immunocytochemistry for APOE (green), MAP2 (violet), and DAPI (blue). (c) Representative western blot of whole lysates showing APOE levels in the three *APOE* genotypes of glutamatergic neurons. β‐ACTIN serves as a loading control.
**Figure S4:** *APOE2* glutamatergic neurons are resistant to irradiation‐induced senescence and DNA damage. (a) Quantification of nuclear size in *APOE* glutamatergic neurons under control conditions and following irradiation‐induced senescence.  ±  represent the mean showing nuclear size distribution across *APOE* genotypes; one‐way ANOVA followed by Tukey's post hoc test, *n* = 3 biological samples per genotype, *p < 0.05. (b) Bar graphs represent the mean ± SEM showing 53BP1 mean intensity *APOE* genotypes in Ngn2 neurons. One‐way ANOVA followed by Tukey's post hoc test; *n* = 6 biological samples per genotype, **p < 0.01, ****p < 0.0001. (c) Representative western blot showing H3K9me3 levels across *APOE* genotypes. β‐ACTIN serves as a loading control. Bar graphs represent the mean ± SEM. One‐way ANOVA followed by Tukey's post hoc test, *n* = 3 biological samples per genotype, *p < 0.05.
**Figure S5:** *APOE2* glutamatergic neurons are resistant to doxorubicin‐induced senescence and DNA damage. (a) Representative images and quantification of the immunocytochemistry for p16 (green), MAP2 (violet), and DAPI (blue). Bar graphs represent the mean ± SEM showing p16 mean intensity across the *APOE* genotypes, *n* = 3, one‐way ANOVA followed by Tukey's post hoc test, ****p < 0.0001. (b) Representative images and quantification of the immunocytochemistry for p‐γH2AX (green), MAP2 (violet), and DAPI (blue). Bar graphs represent the mean ± SEM show p‐γH2AX foci per nucleus across the *APOE* genotypes, *n* = 3, one‐way ANOVA followed by Tukey's post hoc test, *p < 0.05, **p < 0.01, ****p < 0.0001.
**Figure S6:** DNA damage response of *APOE* glutamatergic neurons following doxorubicin‐induced cellular senescence. (a) Representative images and quantification of the immunocytochemistry for 53BP1 (red), MAP2 (violet), and DAPI (blue). Bar graphs represent the mean ± SEM show 53BP1 size of foci across the *APOE* genotypes; *n* = 3, One‐way ANOVA, followed by Tukey's post hoc test, ***p < 0.001, ****p < 0.0001 (b) Representative images and quantification of the immunocytochemistry for p‐ATM (green), MAP2 (violet), and DAPI (blue). Bar graphs represent the mean ± SEM show p‐ATM size of foci across the *APOE* genotypes; *n* = 3, one‐way ANOVA, followed by Tukey's post hoc test, *p < 0.05, **p < 0.01, ***p < 0.001, ****p < 0.0001.
**Figure S7:** *APOE2* NSCs exhibit a more rapid DNA damage response following irradiation. Time‐course of the early DNA damage response to irradiation (5Gy). Representative images and quantification of the immunocytochemistry for: (a) p‐γH2AX (magenta), (b) p‐ATM (magenta), (c) 53BP1 (red) and DAPI (blue). Bar graphs represent the mean ± SEM, *n* = 3; one‐way ANOVA followed by Tukey's post hoc test, *p < 0.05, **p < 0.01.
**Figure S8:** Hippocampus of aged APOE2 knock‐in mice exhibits molecular features associated with longevity. (a) Immunohistochemistry for Hmgb1. Representative images showing Hmgb1 (red) and DAPI (blue) across the *APOE* genotypes. Violin plots show nuclear Hmgb1 mean intensity. One‐way ANOVA followed by Tukey's post hoc test, *n* = 4, *p < 0.05, ****p < 0.0001. (b) Gene set enrichment analysis of Hallmarks of Aging pathways. FDR were calculated using a Fisher's exact test of gene overlap.

**Table S1:** Bulk RNA sequencing of GABAergic neurons derived from iPSCs. Differentially expressed genes *APOE2* versus *APOE4*. Gene Ontology terms enriched for DEGs *APOE2* versus *APOE4*.

**Table S2:** Differentially expressed genes for each cluster identified by Single Cell RNA sequencing of *APOE2* vs. *APOE4* GABAergic neurons.

**Table S3:** Differentially expressed repetitive elements identified using Bulk RNA sequencing from *APOE2* versus *APOE4* GABAergic neurons.

## Data Availability

The data that support the findings of this study are available in NCBIs Gene Expression Omnibus at https://www.ncbi.nlm.nih.gov/geo/. These data were derived from the following resources available in the public domain: GSE143276, https://www.ncbi.nlm.nih.gov/geo/.
