# Supplementary material for: Exceptional Longevity Modifying Allele APOE2 Promotes DNA Signaling Pathways Resisting Cellular Senescence in Human Neurons
Source: Aging Cell. 2026 May 8;25(5):e70494. doi: 10.1111/acel.70494 (PMC13156074; doi:10.1111/acel.70494)

Supplemental Figure 1

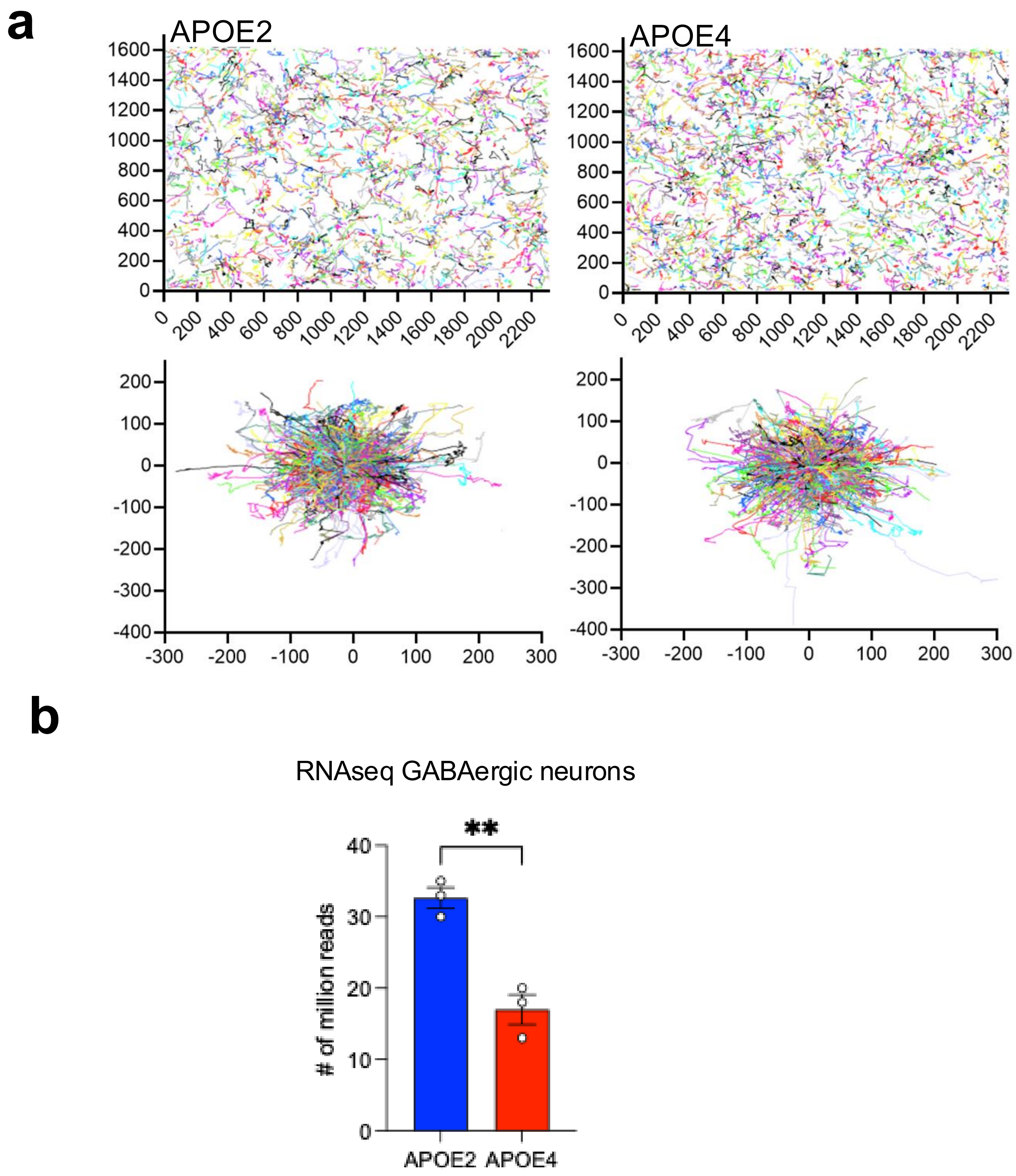

Supplemental Figure 2

a

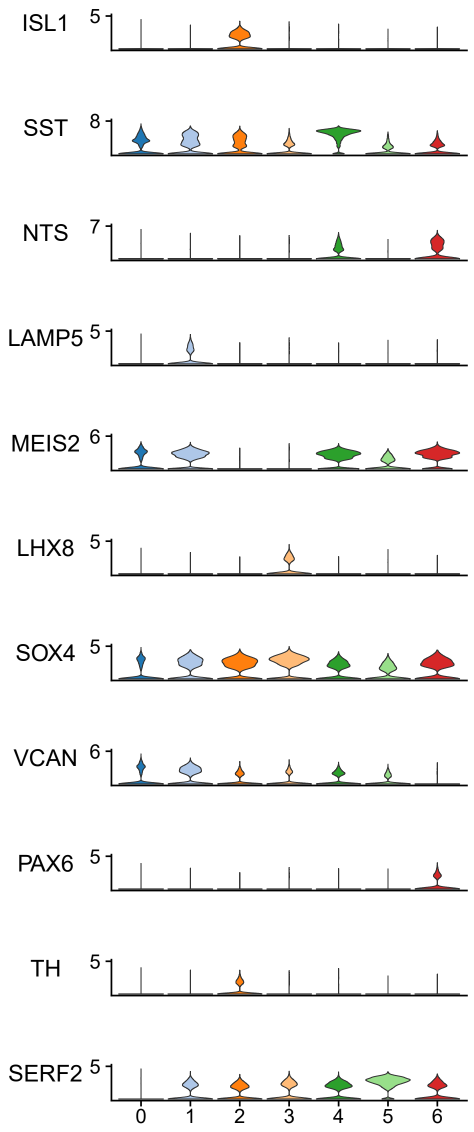

b

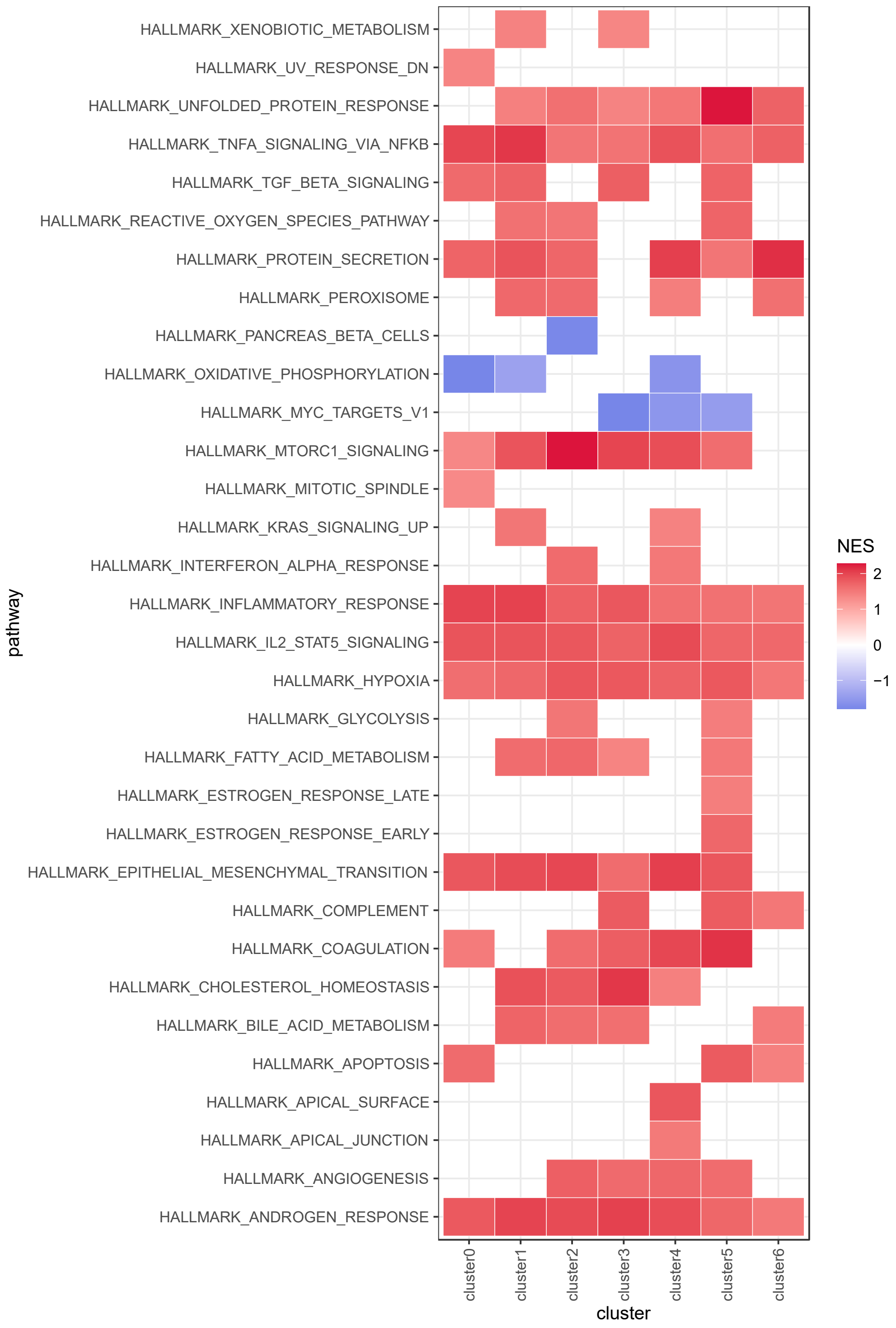

c

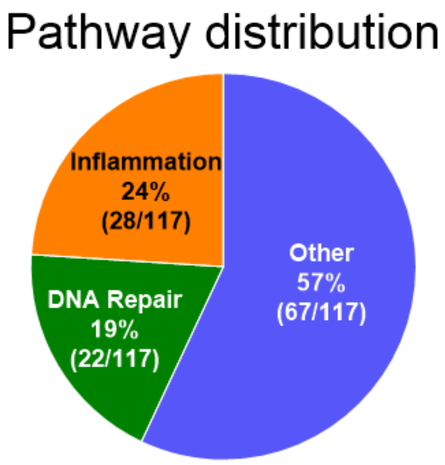

Supplemental Figure 3

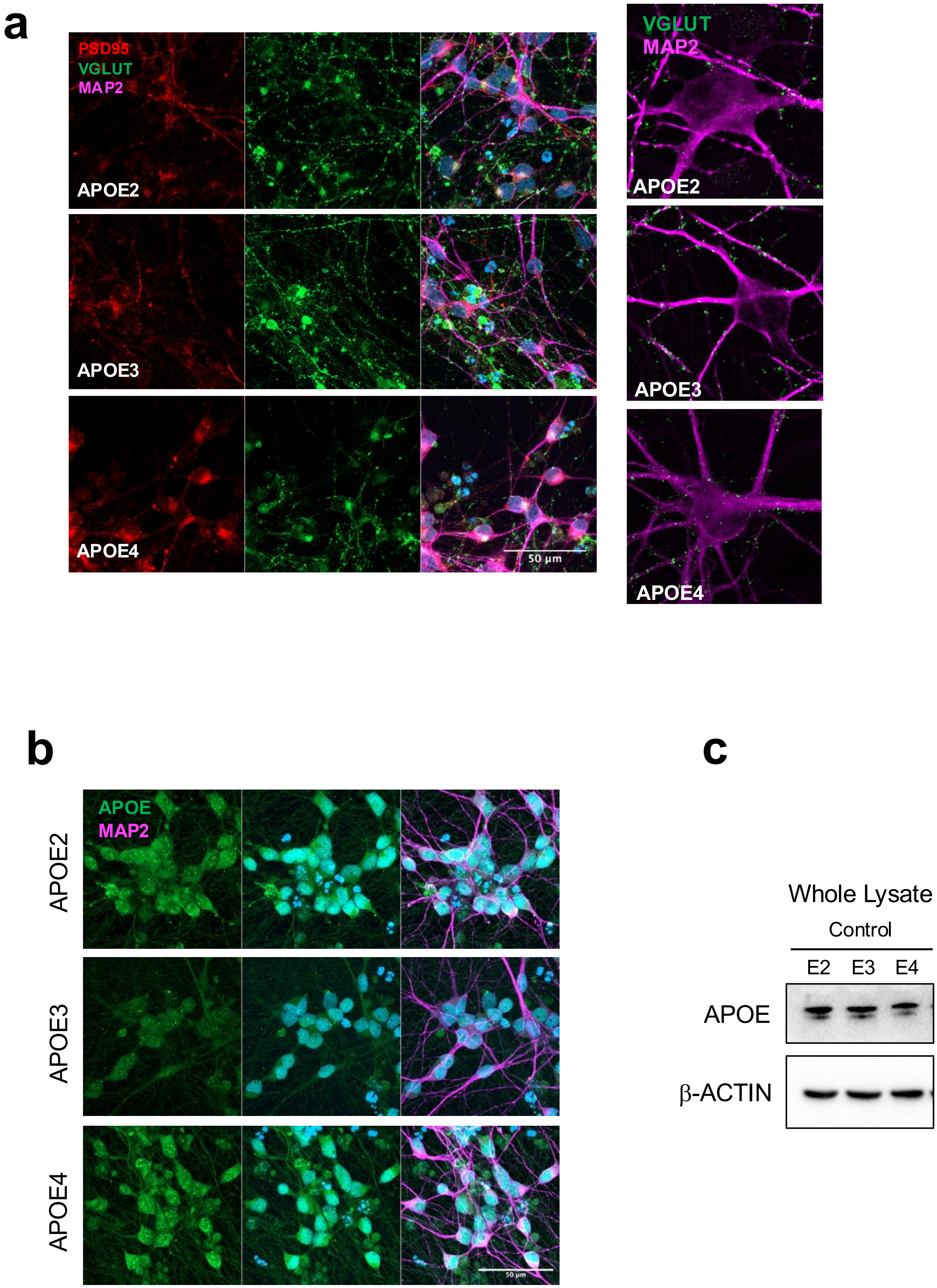

Supplemental Figure 4

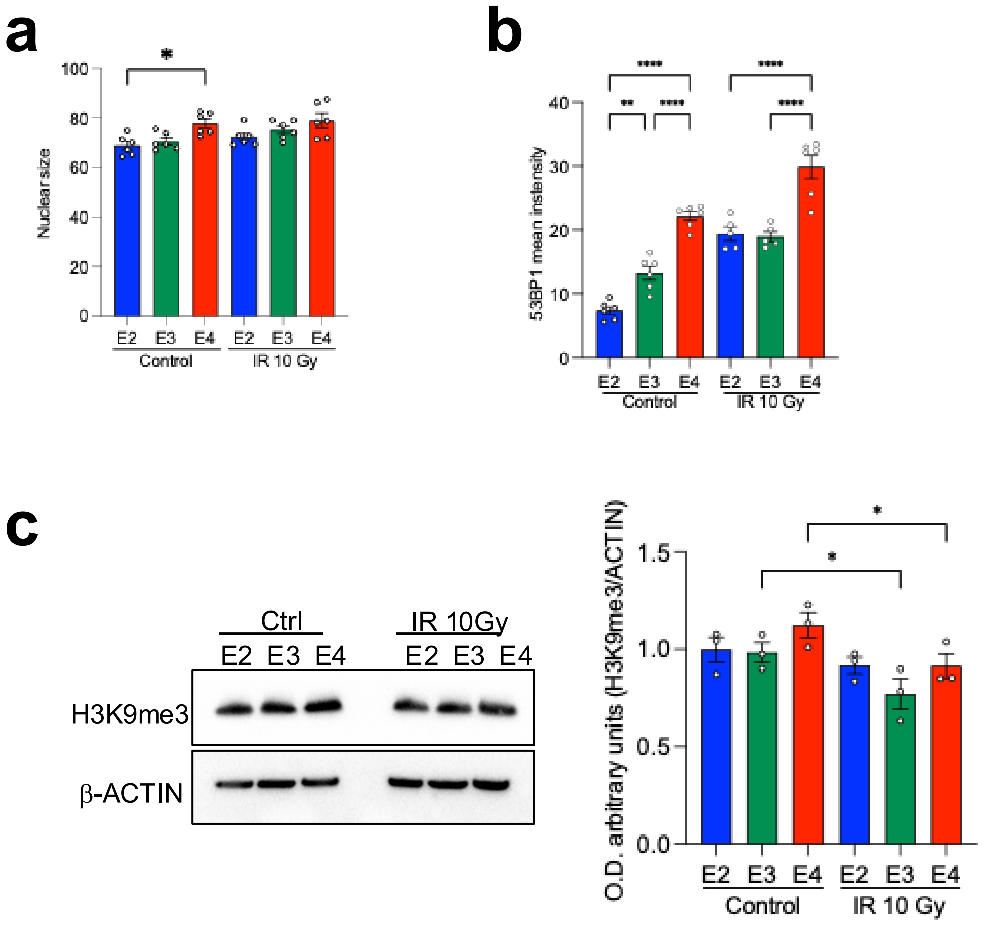

Supplemental Figure 5

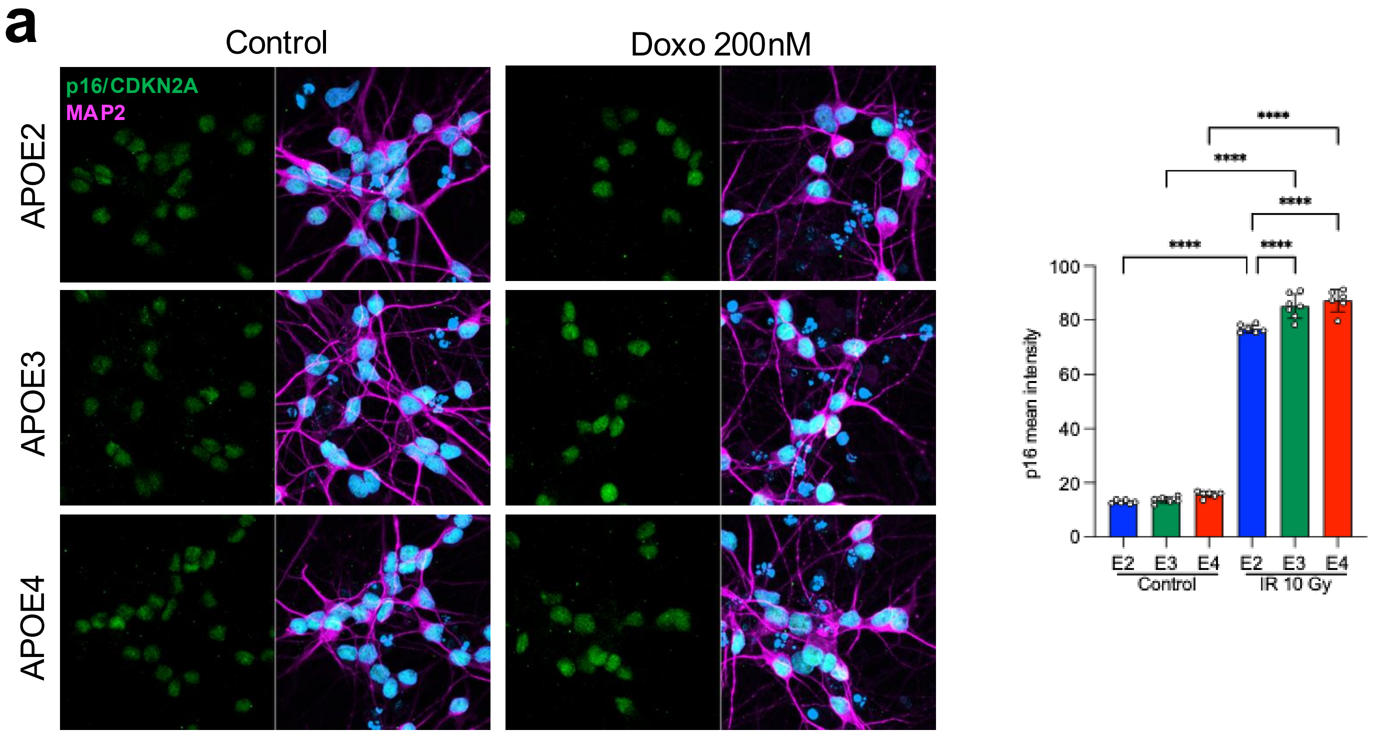

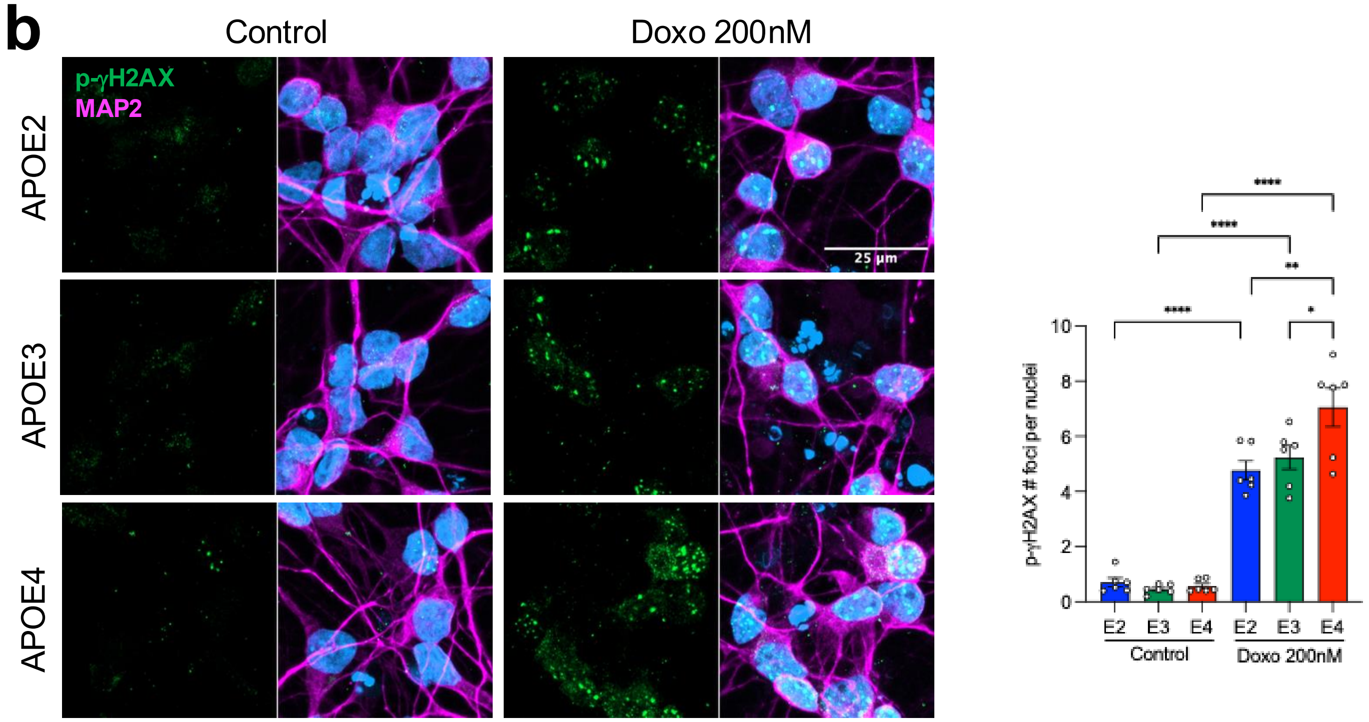

Supplemental Figure 6

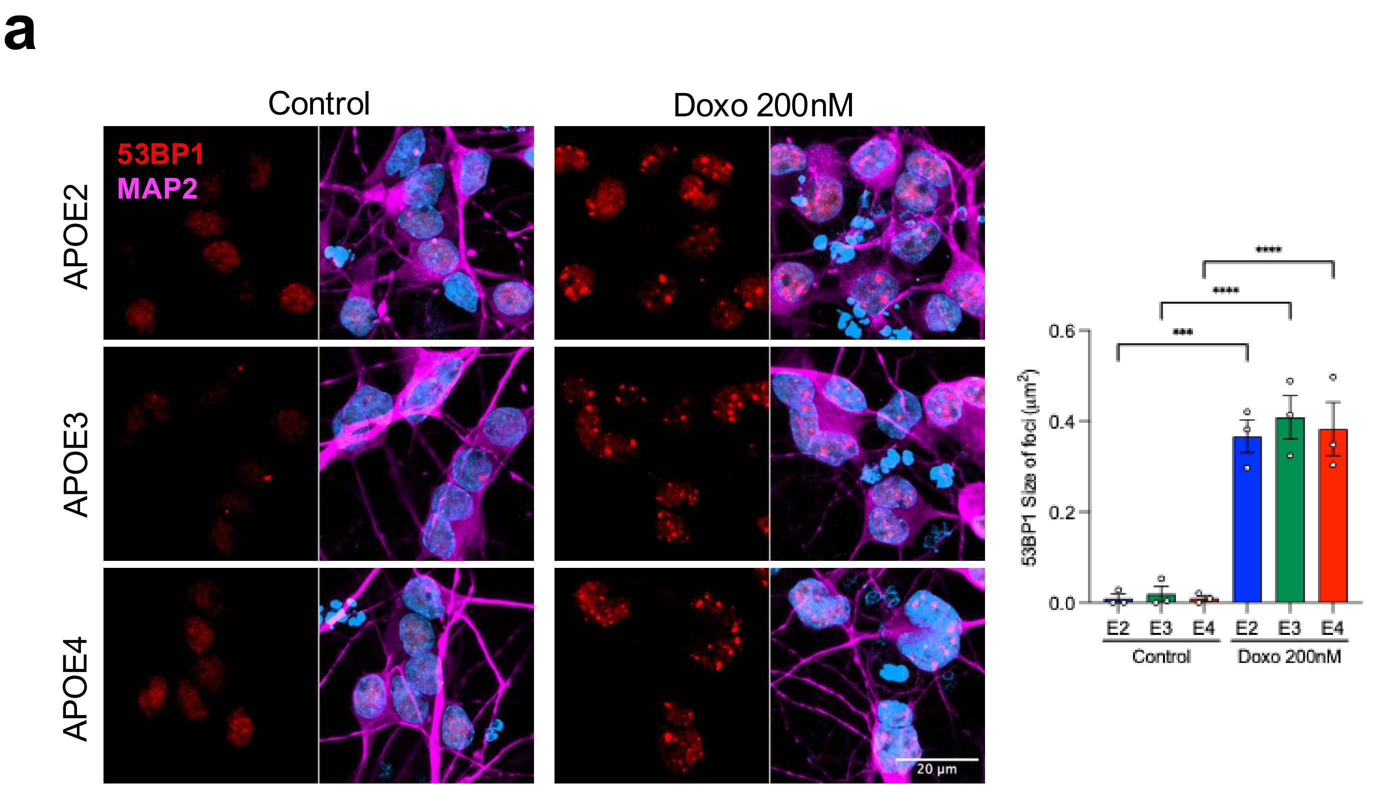

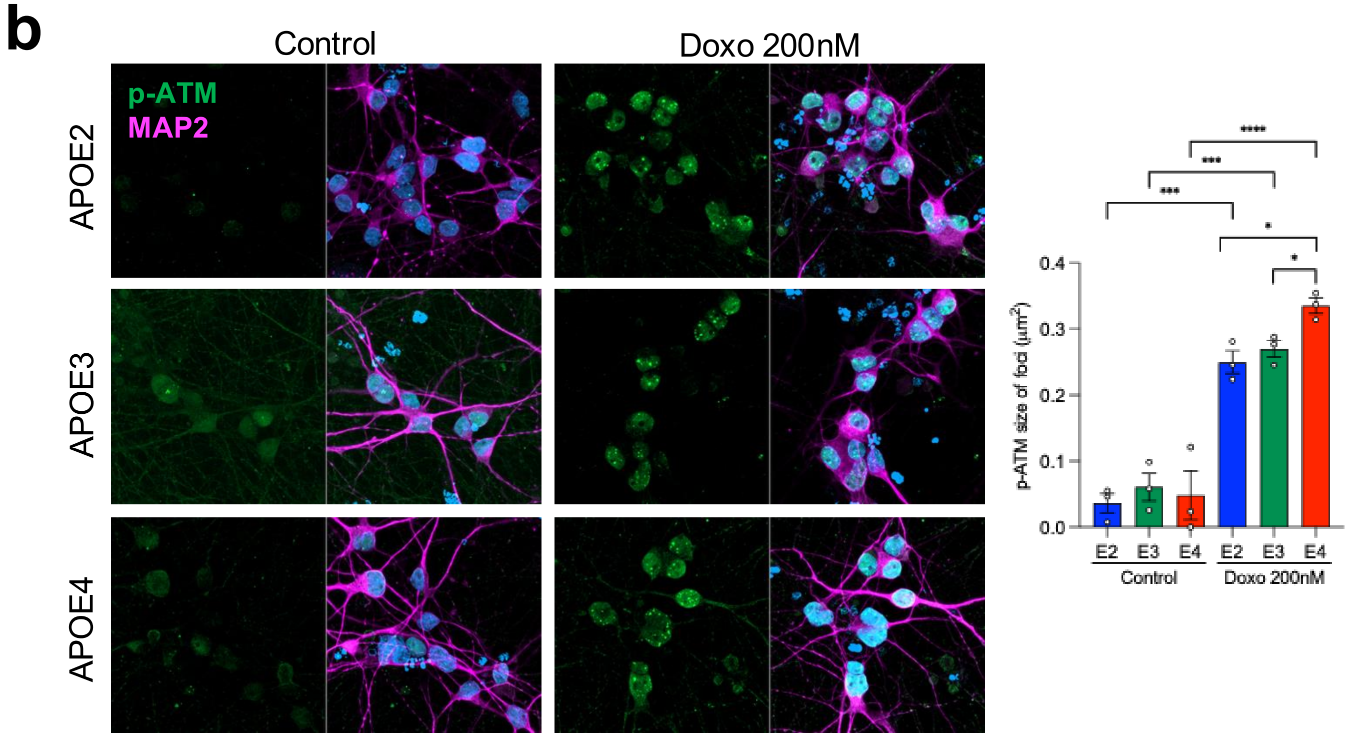

Supplemental Figure 7

a

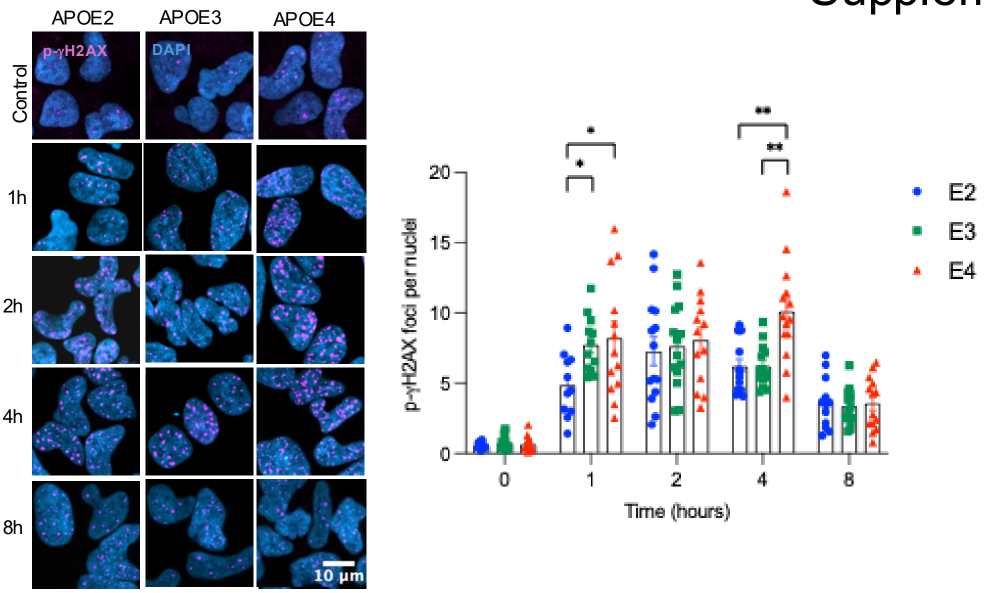

b

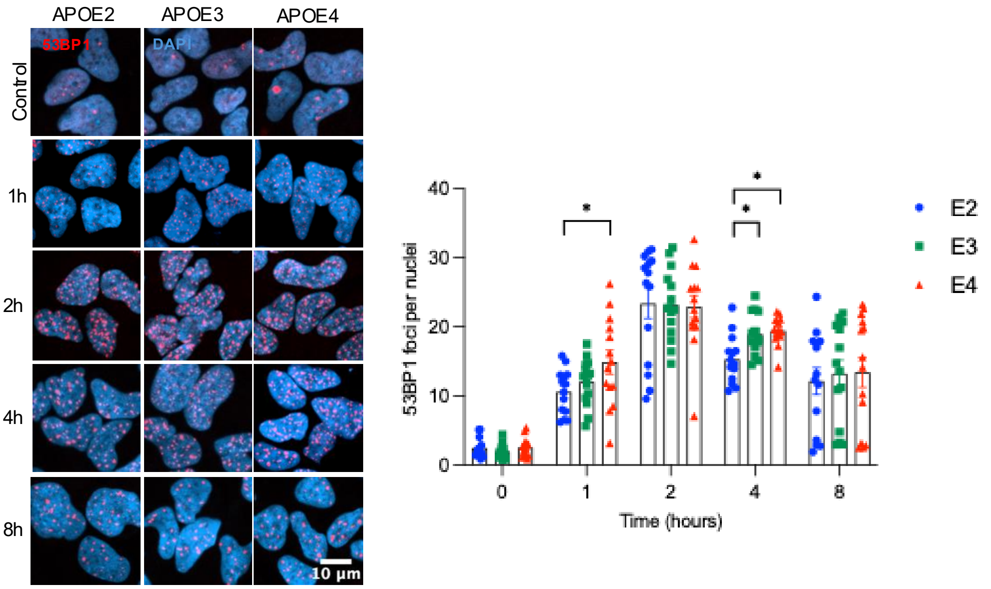

c

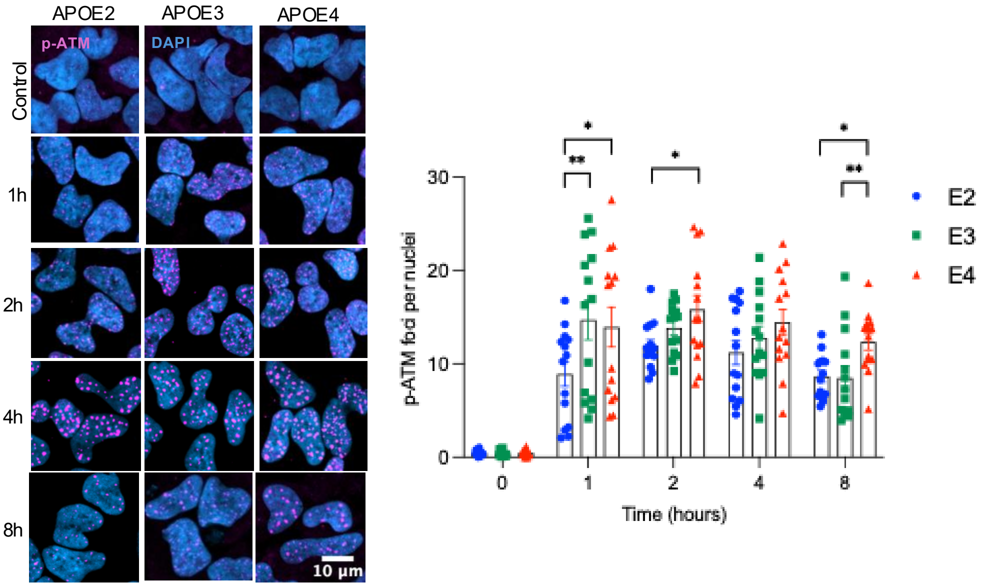

Supplemental Figure 8

a

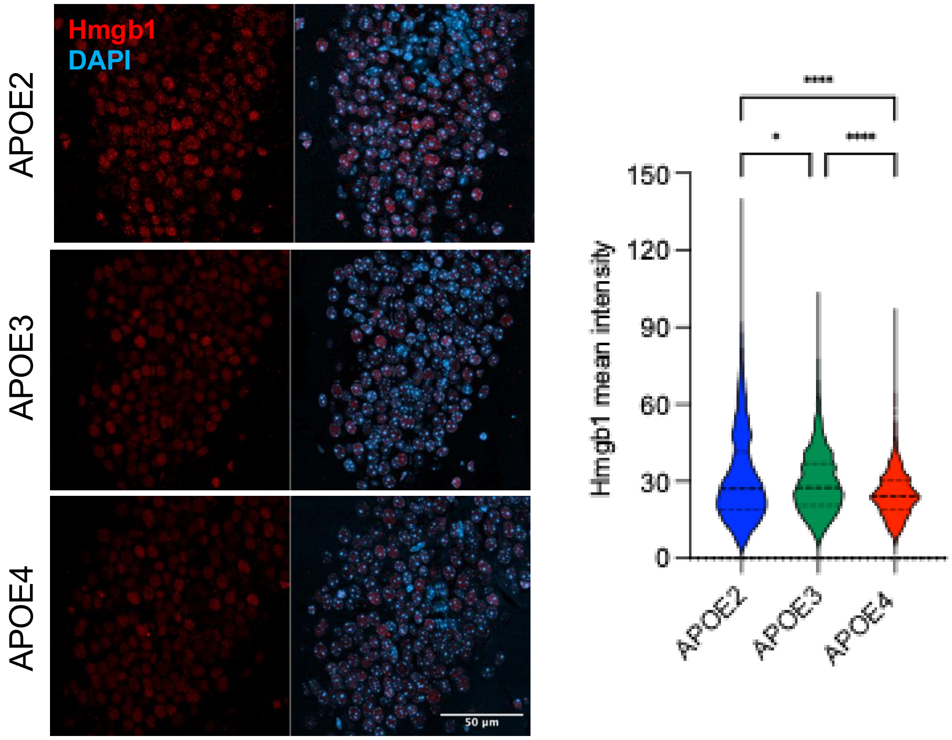

b

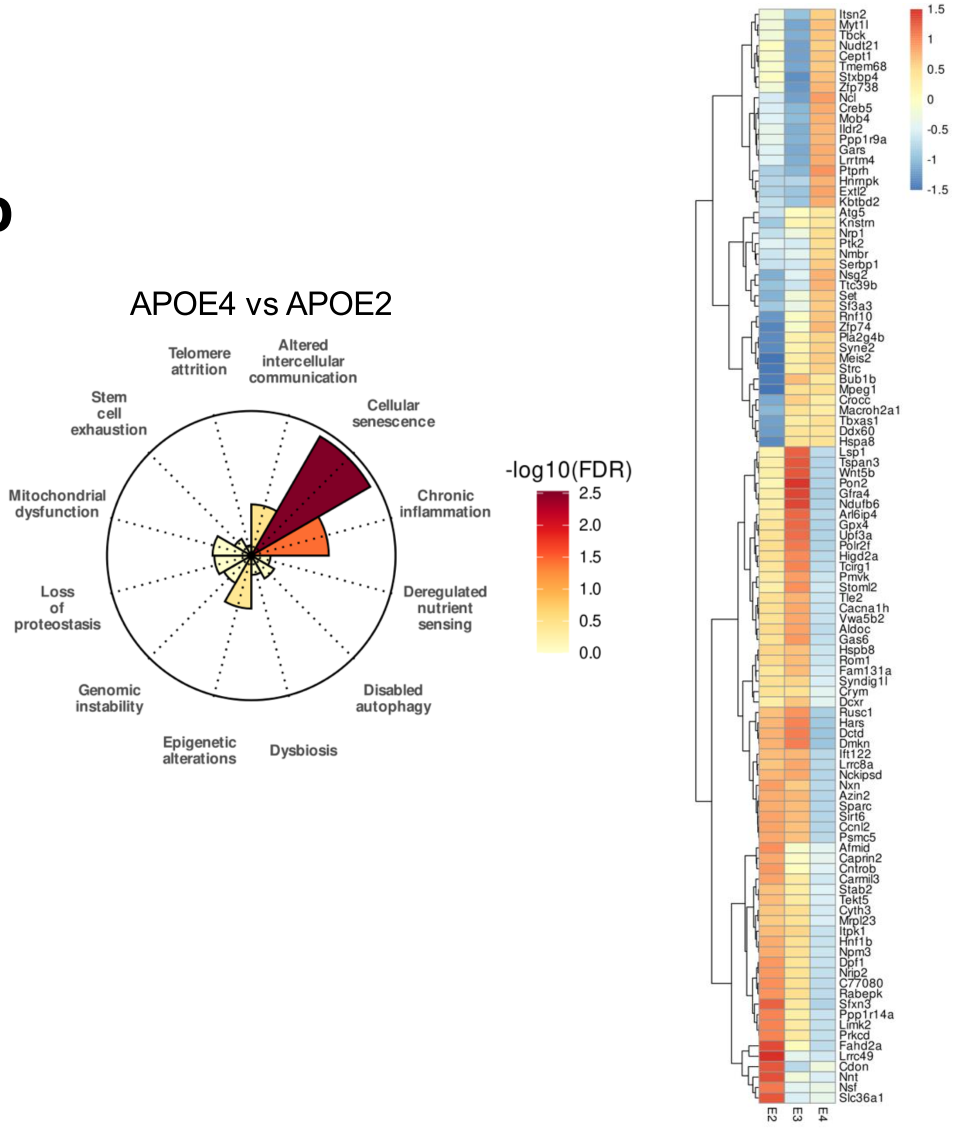

Supplement: Supplementary file 1 — Figure S1: APOE expression and altered motility of GABAergic neurons. (a) Time course of label‐free bright field micrographs of GABAergic neurons recorded and analyzed to quantify cell motility for APOE2 and APOE4 genotypes. Representative movement tracks are shown. Each colored track represents the trajectory of a single identified cell body over a 112‐frame recording, including only cells tracked for at least 10 consecutive frames. Images were taken under brightfield. (b) Differential expression of APOE in GABAergic neurons. Bulk RNA‐seq raw counts of APOE transcripts. Bar plots represent mean ± SEM; t‐test, **p < 0.001, n = 3. Figure S2: Enrichment analysis of APOE GABAergic neurons. (a) Most representative genes in each cluster detected in the single‐cell analysis. (b) Gene Set Enrichment Analysis (GSEA) of Hallmark pathways enriched across the seven clusters identified in APOE GABAergic neurons. Hallmark pathways include TNF‐α signaling, hypoxia, and inflammatory pathways across clusters. Cluster 0 was specifically enriched for mitotic spindle and UV‐induced DNA damage response pathways. (c) Pie chart summarizing pathway distribution across clusters: 24% inflammatory signaling,19% DNA repair, and 57% other pathways. Figure S3: Ngn2 neurons derived from human isogenic iPSCs express markers of fully differentiated glutamatergic neurons across the three APOE genotypes. (a) Representative immunocytochemistry for PSD95 (red), VGLUT (green), MAP2 (violet), and DAPI (blue) with the genotypes indicated. (b) APOE expression in glutamatergic neurons. Representative images of immunocytochemistry for APOE (green), MAP2 (violet), and DAPI (blue). (c) Representative western blot of whole lysates showing APOE levels in the three APOE genotypes of glutamatergic neurons. β‐ACTIN serves as a loading control. Figure S4: APOE2 glutamatergic neurons are resistant to irradiation‐induced senescence and DNA damage. (a) Quantification of nuclear size in APOE glutamatergic neurons under [file ACEL-25-e70494-s002.pdf]
